# Peptide-drug co-assembling: A potent armament against cancer

**DOI:** 10.7150/thno.87356

**Published:** 2023-09-25

**Authors:** Can Wu, Manman Wang, Jinpan Sun, Yongyan Jia, Xiali Zhu, Gaizhi Liu, Yanhui Zhu, Yanbin Guan, Zhenqiang Zhang, Xin Pang

**Affiliations:** 1 School of Pharmacy, Henan University of Traditional Chinese Medicine, Zhengzhou 450046, China.; 2 Academy of Chinese Medicine Science, Henan University of Traditional Chinese Medicine, Zhengzhou 450046, China.

**Keywords:** peptide, co-assembly, nanostructures, drug delivery, cancer therapy

## Abstract

Cancer is still one of the major problems threatening human health and the therapeutical efficacies of available treatment choices are often rather low. Due to their favorable biocompatibility, simplicity of modification, and improved therapeutic efficacy, peptide-based self-assembled delivery systems have undergone significant evolution. Physical encapsulation and covalent conjugation are two common approaches to load drugs for peptide assembly-based delivery, which are always associated with drug leaks in the blood circulation system or changed pharmacological activities, respectively. To overcome these difficulties, a more elegant peptide-based assembly strategy is desired. Notably, peptide-mediated co-assembly with drug molecules provides a new method for constructing nanomaterials with improved versatility and structural stability. The co-assembly strategy can be used to design various nanostructures for cancer therapy, such as nanotubes, nanofibrils, hydrogels, and nanovesicles. Recently, these co-assembled nanostructures have gained tremendous attention for their unique superiorities in tumor therapy. This article describes the classification of assembled peptides, driving forces for co-assembly, and specifically, the design methodologies for various drug molecules in co-assembly. It also highlights recent research on peptide-mediated co-assembled delivery systems for cancer therapy. Finally, it summarizes the pros and cons of co-assembly in cancer therapy and offers some suggestions for conquering the challenges in this field.

## 1. Introduction

Cancer is still one of the main problems threatening human health while its available treatment method, such as chemotherapy, radiotherapy, immunotherapy and phototherapy are unsatisfactory and have major side effects 1, 2. Even though there have been significant advances in cancer treatment, it is still not yet possible to entirely eradicate the disease. With the development of nanomedicine, new nano drug delivery systems, such as nanotubes, nanofibers, micelles and nanoparticles, have made significant progress in enhancing the therapeutic effects, reducing the side effects, and increasing the solubility of anticancer drugs 3, 4. Although the use of nano drug delivery systems has improved, there are still problems that need to be fixed. For instance, synthetic inorganic materials may be associated with poor biocompatibility and unavoidable immunological responses 5. Besides, the application of synthetic polymers is limited by the complex functionalization methods and bounded capacity to deliver protein drugs 6. In addition, due to the complexity of the tumor microenvironment, the single targeting capability of nanodrugs cannot satisfy the requirement for precise drug delivery 7. Therefore, it is imperative to create a delivery system that is both non-toxic and highly efficient, as well as possessing superior targeting capabilities.

**Figure 1:**
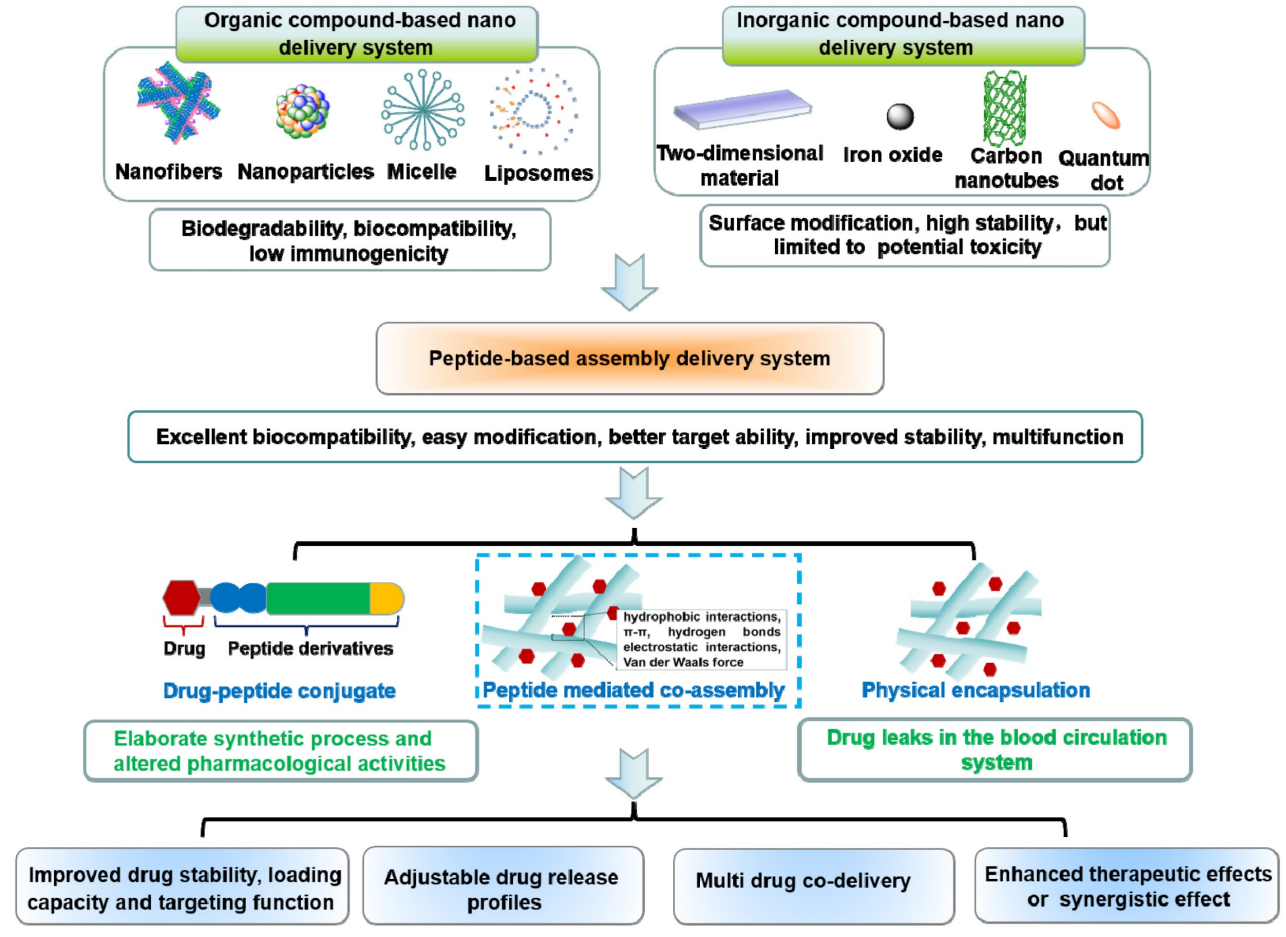
Three types of peptide-based assembly delivery system: (I) Drug-peptide conjugates; (II) Peptide mediated co-assembly; (III) Physical encapsulation.

Peptides are a class of compounds containing two or more amino acids linked by amide bonds, which widely exist in the human body and exerts specific biological functions. Peptide self-assembly involves non-covalent interactions that facilitate the spontaneous formation of well-ordered architectures 8, 9. The superior performance of peptide-based self-assembly systems has garnered considerable attention, with notable advantages such as excellent biocompatibility, simple modification, better scalability, and enhanced stability 10, 11. Self-assembling peptides themselves exhibits excellent anti-cancer and antibacterial properties 12-14. Moreover, self-assembling peptides have been exploited as drug delivery carrier for multiple biological applications, such as anti-bacterial, anti-inflammatory and tissue engineering 15-17. In particular, self-assembled peptide drug delivery holds great promise for treating cancer due to its multifunctionality 18. In general, physical encapsulation or covalent conjugation can be used to load medicinal molecules into peptides. However, the therapeutic effects of physical encapsulation may be undesirable due to drug leaks in the blood circulation system, and covalent conjugation is challenged by the elaborate synthetic process and altered pharmacological activities. The synthesis of peptide-drug conjugates requires not only solid-phase synthesis, but also complex liquid phase synthesis, as well as subsequent purification steps 19-22. Besides, even minute adjustments in the structure of peptide-drug covalent conjugates can pose a significant impact on the performance of the system. In ideal conditions, pharmacological activity is increased, but sometimes the opposite results are also seen (**Figure 1**) 23-24. To solve these problems, a more elegant peptide-based assembly strategy should be developed.

Notably, peptide-mediated co-assembly with drug molecules provides a new method for constructing nanomaterials with structural complexity and stability. Stable co-assembled supramolecular nanostructures are made possible by the noncovalent interactions, which often include hydrogen bonds, π-π stacking, electrostatic interactions, hydrophobic interactions, and van der Waals forces 25, 26. The co-assembly strategy can be used to design various nanostructures for cancer therapy, such as nanotubes, nanofibrils, hydrogels, and nanovesicles. Recently, co-assembled nanostructures have gained tremendous attention due to their exceptional advantages in cancer treatment 27-29. Compared with other delivery systems, the advantages of theses co-assembled nanostructures includes the following aspects: (1) In contrast to simple encapsulation, the stability of the drug can be improved and drug leakage can be avoided by the intermolecular interaction between peptide and drug molecules; (2) Adjustable drug release profiles can be obtained by changing the ration of peptide and drug molecules; (3) The drug loading capacity can be significantly improved through the non-covalent interaction between the residues of peptide and drug molecules; (4) Therapeutic effects of the drug can be enhanced by utilizing multifunctional peptide with excellent targetability; (5) Multiple drug molecule can be simultaneously loaded in co-assembled nanostructures for combination therapy (Figure 1). Comprehensively, these superiorities provide a wide latitude for the development of peptide-based co-assembled nanostructures for cancer therapy.

**Figure 2:**
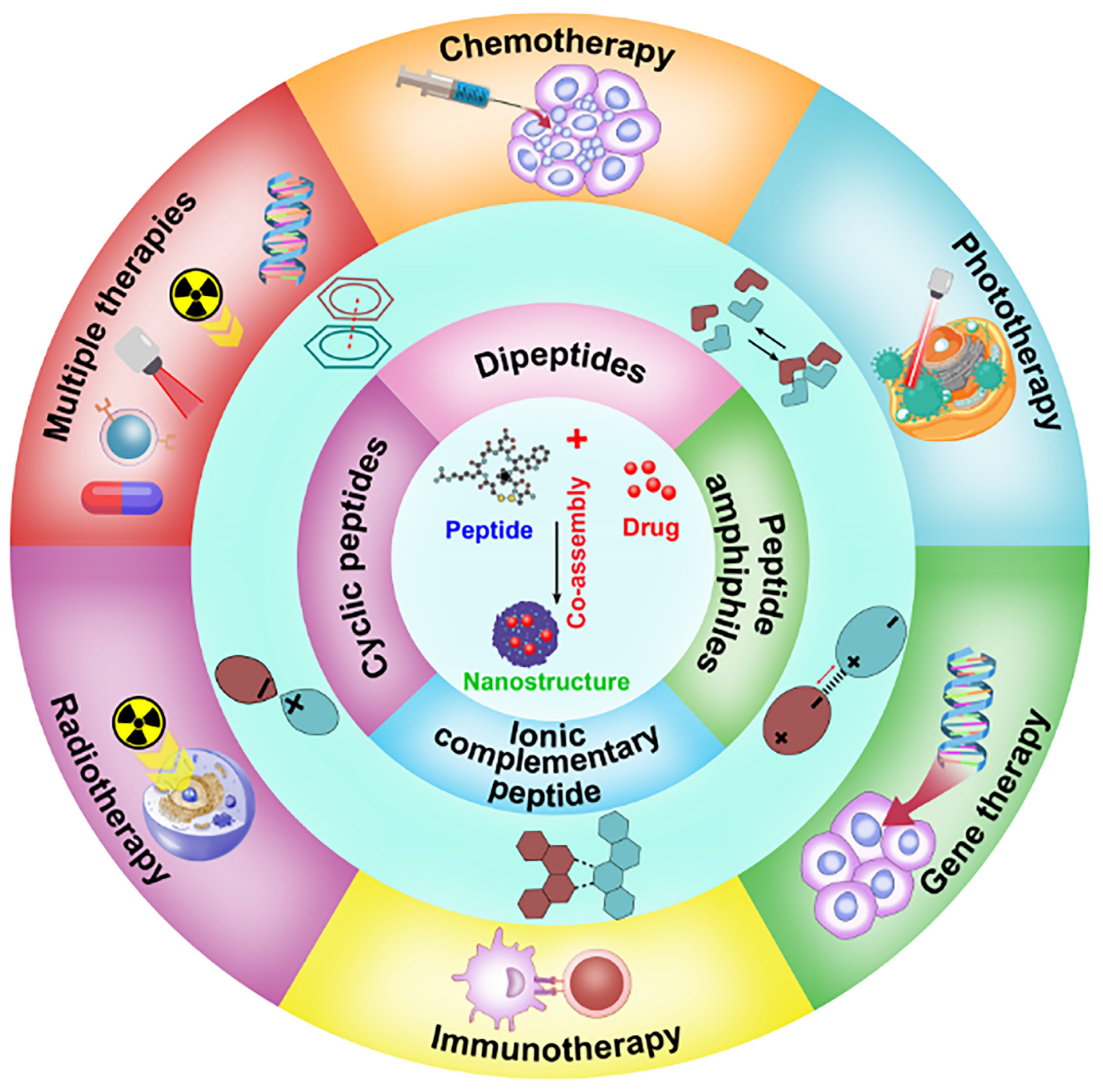
Schematic illustration of co-assembled peptide-drug nanomaterials in various cancer therapeutics.

In this review, we summarized the recent research progress on the co-assembled peptides-drug molecule for cancer therapy, including the classification of assembled peptides, driving forces for co-assembly, and specifically, the recent progress in the co-assembly of peptides and drug molecule (Figure 2). We anticipate encouraging opportunities for developing intricate peptide-drug co-assembled nanostructures that offer effectivecancer therapies and support the comprehension and clinical implementation of these nanostructures through this review.

## 2. Classification of building blocks of assembled peptides

Peptides are compounds with long chain structures composed of polar or non-polar amino acids. According to the various constituent amino acids and different motifs, the building blocks of assembled peptides used for co-assembly can be divided into aromatic dipeptides, peptide amphiphiles, ion complementary peptides and cyclic peptides.

### 2.1. Aromatic dipeptides

Phenylalanine is the simplest building block commonly used in the construction of self-assembled peptides. Phenylalanine dipeptide (FF) is the core recognition sequence present in Alzheimer's amyloid β-peptide. Phenylalanine dipeptide and its derivatives are always used to build supramolecular materials with various structures *via* π-π stacking and hydrogen bonding interaction 30. Gazit and his colleagues revealed that FF self-assemble to form 1D nanotubes with specific structures, which obtained extensive attention in the field of supramolecular peptide-based self-assembly 31, 32. Afterward, Ulijn and his colleagues carried out systematic research on tripeptides-based self-assembly by utilizing computer simulation experiments 33. Afterwards, various experiments have demonstrated the morphology of peptide-based self-assembled nanostructure can change from 1D (fiber and tube) to 2D (sheet) by adjusting the sequence of dipeptides derivatives.

**Figure 3:**
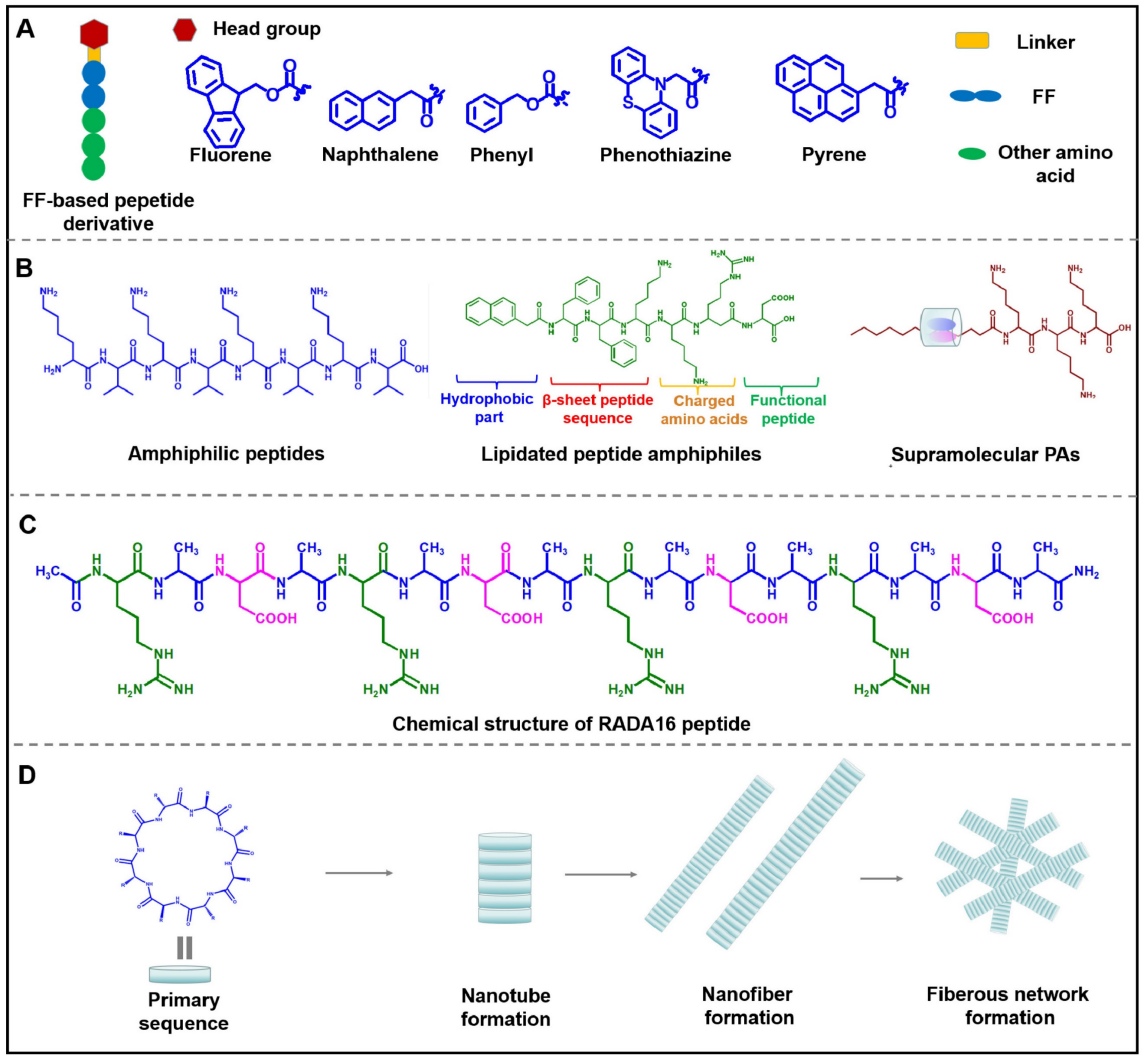
Structure diagram of various building blocks of assembled peptides. (A) Structure diagram of FF-based peptide derivative. (B) Three types of peptide amphiphiles: Amphiphilic peptides, lipidated peptide amphiphiles, and supramolecular PAs. (C) Chemical structures of ionic-complementary peptide RADA16. (D) Assembly process of cyclic peptide.

In recent studies with diphenylalanine, it has been demonstrated that FF-based self-assembled nanomaterials exhibited enhanced stability, structural flexibility and accessibility. Besides, the modification of the functional group at the N-terminal or C-terminal of the peptide can not only change the assembly behavior of the peptide, but also endow the peptide with multifunction. The N-terminal dipeptide could be modified by introducing aromatic groups such as naphthylacetic acid (Nap), tert-butoxycarbonyl (Boc), and 9-Fluorenylmethoxycarbonyl (Fmoc) (Figure 3A) 34-36. Duan et al. designed and synthesized D-Nap-GFFY-T317, which could be self-assembled into nanofibers to effectively inhibit the proliferation of tumor cells 37. As the research deepens, FF and its derivatives are expected to be employed as multifunctional nanomaterials with varied performance. For example, carboxybenzyl-protected diphenylalanine (ZFF) could co-assemble with different bipyridine derivatives to form various supramolecular nanostructures through intermolecular hydrogen bonding 38. Besides, different functional groups, such as targeting sequence, specific stimuli-responsive sequence and therapeutic sequence, can be introduced into the C-terminal of the peptide to construct multi-functional nanostructures. Overall, FF is the most favorable peptide building block due to its structural simplicity, functional diversity, and cost-effectiveness. Various supramolecular structures such as nanofiber, nanotubes and ribbons can be obtained by utilizing the self-assembly of FF *via* the interactions of hydrogen bonding and π-π stacking 39-44. Many research groups at home and abroad have also successively studied FF-based self-assembly and explored its potential applications in drug delivery, tissue engineering, and antibacterial 45-49. With the deepening research of FF-based assembly, it can be expected that introducing multifunctional modules into peptides will provide an effective way to obtain nanomaterials with improved intrinsic properties and sophisticated functions, which ultimately realize the potential applications in nanomedicine.

### 2.2. Peptide amphiphiles

Peptide amphiphiles (PA) are amphiphilic peptides that prefer to form highly defined nanostructures in a particular situation. Peptide amphiphiles can usually be classified into three types: (1) Amphiphilic peptides; (2) Lipidated peptide amphiphiles; (3) Supramolecular PAs 50-53. Generally, PA can self-assemble into varied nanostructures such as nanofibers, nanotubes and micelles under the balanced intramolecular and intermolecular interactions of amino acid residues. The amphiphilic characteristics inherited by PA enable it assemble into nanostructures with a hydrophobic core and highly hydrated and charged surface. Drug molecules can be loaded in the different parts of PA-based assembled nanostructures. In general, lipophilic drugs molecules can be loaded in the hydrophobic part, while water-solubility drugs molecules usually are encapsulated in the hydrophilic surface.

Amphiphilic peptides are a representative class of PA, composed of pure polar and non-polar amino acids. For example, V_6_K_3_ is a positively charged amphiphilic peptide, which exhibited a morphological transformation from nanoparticles to nanofibers in the presence of plasma amine oxidase and could be exploited as an ideal carrier for delivery lipophilic anticancer drugs 54. Among all PA, lipidated peptide amphiphiles are the most representatives PA, which usually consists of four essential domains as shown in Figure 3B: (1) hydrophobic domain, such as alkyl chain, fluorene methoxycarbonyl (Fmoc) or 2-naphthylacetic acid (Nap); (2) β-sheet peptide sequence for promoting peptide self-assembly; (3) Negatively charged or positively amino acids, which endow peptide with a certain solubility in water; (4) functional peptide, such as cell adhesion peptides and targeting peptide 55, 56. During the process of self-assembly of lipidated peptide amphiphiles, the hydrophobic domain can form internal hydrophobic nuclei, while the functional part is exposed to the surface of the nanostructure. β-sheet sequence and charged amino acids paly an indispensable role in regulating the stability and solubility of peptides. Lipidated peptide amphiphiles tend to form 3D nanofibers, and then the fibers gradually elongate to form hydrogels. Lipidated peptide amphiphiles have demonstrated considerable application prospects in the fields of drug delivery, anti-bacterial therapies, and regenerative medicine thanks to their superior ability to form structurally ordered nanostructures. Supramolecular PAs are obtained by integrating supramolecular chemistry during the preparation process. In brief, two separate molecules are linked by covalent bonds or supramolecular interactions. Debapratim et al designed a ternary complex by using naphthalene functionalized peptide (Nap-P), cucurbit[8]uril (CB[8]) and a viologen unit (MV-CHO). The ternary complex formed a transient supramolecular PA when dodecylamine (DA) exists under basic conditions 57, 58. Thus, various supramolecular PA with structural flexibility and stimulus responsiveness can be obtained by using this approach. In general, although amphiphilic peptides-based nanomaterials have the virtue of easy preparation, structural flexibility and biocompatibility, amphiphilic peptides-based complicated structures need to be exploited for developing advanced nanomaterials.

### 2.3 Ionic-Complementary peptide

Ion complementary peptide is another common building block for developing supramolecular nanomaterials, which contains an alternating arrangement of charged amino acid and hydrophobic amino acids residues 59, 60. The assembly of this peptide relies on the formation of hydrogen bonds, which tend to form β-sheet structures with two different polar and nonpolar surfaces. The polar surface of the β-sheet structure is composed of positively and negatively charged amino acids, and the non-polar surface contains hydrophobic amino acids. The structure of alternate amino acid residues boosts the process of assembly, inducing the formation of the hydrophobic core and hydrophilic surface of nanofiber when the two β-sheet are stacked into a basic fibril unit.

RADA16 is a representative ionic-complementary peptide, which is composed of 16 amino acids, including positively charged arginine (R), hydrophobic alanine (A), and negatively charged aspartic acid (D) (Figure 3C) 61, 62. More and more research indicated that the modification of RADA16 could endow this peptide with novel functions. Tao et al. designed two functionalized peptides, namely CRP ((RADA)_4_-GGQQLK) and CBP ((RADA)_4_-GSVLGYIQIR). These two peptides were regarded as Ca^2+^ binding peptides, which could self-assemble into nanofibers and crosswise link to form meshlike networks when being exposed to Ca^2+^. Then, blood coagulation factor XIIIa could further trigger the network to form a compact nano-band-aid to stop bleeding 63. By conjugating HS-RADA16-GG-melittin and 4-arm PEG-Maleimide, Jin et al. synthesized MRP, which was used to encapsulate the ultrafiltration retentate of irradiated tumor cells (UF) to obtain UF@MRP for synergistically killing tumor cells 64. Later results showed that RADA16 was capable to form nanofibrous hydrogel for delivering exogenous DCs, antigens and anti-PD-1 antibodies for immunotherapy against cancer 65. In generals, RADA16 and its derivates can self-assemble into nanofibers, which exhibit good biocompatibility and negligible immunogenicity. The further study focuses on the RADA16 derivates with longer sequences that will promote the development of ionic-complementary peptide-based nanostructures with more flexible structures and stronger mechanical properties.

### 2.4. Cyclic peptides

In 1974, De Santis et al. profoundly summarized that cyclic peptide consisted of a certain number of alternate D and L-amino acids, which could self-assemble into rigid cylindrical nanotubes with β-sheet structure under non-covalent interaction (Figure 3D) 66. In the 1990s, Ghadiri and his colleagues designed cyclic peptides and first reported the self-assembled nanotube structure of the cyclic peptide 67. Follow-up released research comprehensively studied the properties of cyclic peptides 68-70. Compared with linear peptides, cyclic peptides exhibited several advantages:(1) High stability and reduced degradation of proteases; (2) Simplicity of transmembrane transport. Given the favorable properties of cyclic peptides, many artificial cyclic peptides have been regarded as therapeutic agents and drug delivery carriers. For example, Gao and co-works designed a novel cyclic peptide C25, which could target the immune checkpoint by blocking the interaction of LAG-3 and HLA-DR, leading to promising anticancer effects 71. Niu and co-works designed a glutathione (GSH)-responsive cyclic peptide (C-1), which could convert into a linear peptide under the overexpressed GSH in cell and further self-assembled into nanofibers, leading to caspase-3 activation and cell death 72. Schmuck et al. reported a cyclic peptide which was conjugated with guanidiniocarbonyl pyrrole (GCP) residue. The GCP-containing cyclic peptide could assembled into stable nanostructure for delivering DNA into cells 73. Functionalized cyclic peptides also can be used for enhancing the targeting ability of drugs. Gellerman et al. designed a novel somatostatin-derived cyclic peptides-drug conjugates, which exhibited preferable targetability towards pancreatic cancer cells with overexpressed somatostatin receptors 74. All in all, thanks to the unique advantages of cyclic peptides, such as versatility, targetability and structural stability, it has been developed as an efficient carrier for the delivery of small-molecule and macromolecule therapeutic agents.

## 3. Driving forces for co-assembly

**Figure 4:**
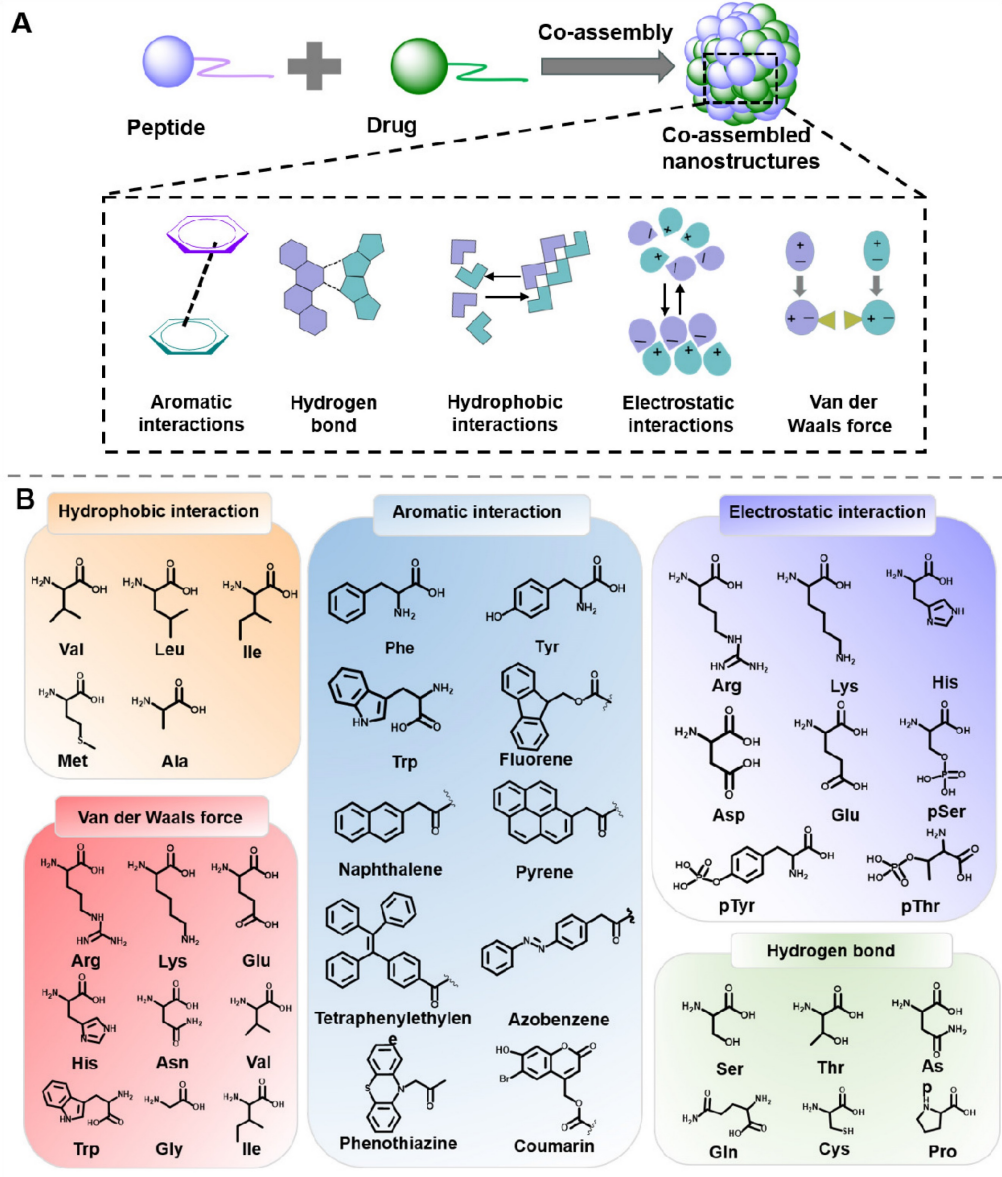
(A) Driving forces involved in peptide-based co-assembly: Aromatic interactions, hydrogen bond, hydrophobic interactions, electrostatic interactions and Van der Waals force. (B) Commonly used amino acids and molecules in peptide-based co-assembly.

Multiple nanomaterials can be designed through the co-assembly of peptide and drug molecules, which exhibit considerable advantages, such as enhanced stability and prolonged retention time. The co-assembly process relies on the balance of intermolecular noncovalent force between peptide and drug molecules, including hydrophobic interactions, aromatic interactions, electrostatic interactions, hydrogen bonds, and Van der Waals force (Figure 4) 75, 76.

### 3.1. Hydrophobic interactions

Hydrophobic interactions is one of the main driving forces for assembly, which are generate by the hydrophobic amino acids residues in hydrophobic conditions. Amphiphilic molecules in water tend to form micelles rather than nanofibers when the hydrophobic interactions are the only self-assembly force 77, 78. Hydrophobic parts of amphiphilic molecules are prone to gather to form inner cores when the hydrophilic parts are exposed to water. Significantly, this hydrophobic interaction is essentially ascribed to the entropy effect, not the enthalpy effect. Most notably, there are always several driving forces at work in a co-assembly system, and the main dominant interactions are always associated with the peptide residues and the drug molecule structure. For instance, Chen et al. designed an ionic-complementary peptide EAR8-II, which could co-assemble with liposoluble anticancer drug pirarubicin (THP) into stable EAR8-II-THP nanofiber in aqueous media 79. Further results indicated that the hydrophobic interactions between aromatic rings of THP and alanine residues in EAR8-II were the dominant forces for the formation of the co-assembled EAR8-II-THP nanocomplexes. Thus, the construction of a nanoplatform by utilizing the hydrophobic interaction between peptide and drug molecules may be a feasible strategy for hydrophobic drugs delivery.

### 3.2 Aromatic interactions

 π-π stacking is one of the main forces of self-assembly and co-assembly of peptides, especially for peptides containing aromatic groups. As a result of the peptides with aromatic groups' low solubility, π-π stacking is quite noticeable in water. When the assembly occurs in organic solvents, the aromatic interactions will function as the unique driving force. Domestic and foreign research groups represented by Gazit et al. have demonstrated that diphenylalanine peptide (L-Phe-L-Phe) could self-assemble into an ordered tubular structure under the π-π interaction and hydrogen bond 80. Besides, for aromatic groups-containing peptides and drugs, π-π stacking would be the main force for promoting and stabilizing the co-assembled peptide-drug complexes. For example, Nilsson et al. designed several Fmoc-Phe-DAP molecules, which were composed of Fmoc-Phe and diaminopropane. The Fmoc-Phe-DAP could self-assemble into hydrogels under the impact of sodium chloride, which was suitable for delivering diclofenac. *In vitro* release experiments revealed that the attractive π-π interactions between peptide and diclofenac played an important role in the release amount of diclofenac 81. Based on the research, thanks to the stable π-π interactions, aromatic peptides would be considerable building blocks for constructing nanoplatforms for delivering drugs containing aromatic groups.

### 3.3 Electrostatic interactions

Electrostatic interaction is generally not directional but is another important interaction for prompting assembly. Research suggested that the shielded Coulomb effect between positive and negative charges would trigger the formation of ion pairs. However, the electrostatic interaction is always influenced by environmental conditions, which will be weakened by hydrated ions and the charge shielding effect from salt 82. In the assembly process of charged peptides, the electrostatic interaction plays an unsubstitutable role for regulating the assembly behavior. In 1993, Rich and his colleagues discovered the simple self-complementary EAK16 peptide, which consisted of oppositely charged amino acids. EAK16 could spontaneously assemble to form a fairly stable macroscopic membrane 83. Electrostatic interaction also plays an indispensable role in the co-assembly of peptides and peptides, or peptides and drugs. For example, Webber et al. designed tetrapeptides (KWKW, DWDW) with opposite charge. The electrostatic interactions between DWDW and KWKW induced the hierarchical structures with favorable mechanical properties 84. Zhong et al. designed a series of novel peptide derivatives 1-RGDH_n_ (Nap-GFFYGRGDH_n_), which could co-assemble with doxorubicin (DOX) to confers 1-RGDH_n_ hydrogels under electrostatic interactions 85. In a similar way, charged peptides could be utilized to design various carriers for drug delivery with complementary charges in the future.

### 3.4 Hydrogen bond

Hydrogen-bond is considered crucial for stabilizing the structural organization of protein and peptide. Peptide possesses hydrogen bond formation sites, such as carboxyl, and amide groups in the side chains and backbone of the peptides. When there are strong hydrogen bonds in the system, peptide tends to form β-sheet structures. However, the strength of the hydrogen bond can be significantly influenced by the direction and distance between hydrogen acceptor and donor pairs. Diphenylalanine prefers to form nanotubes in an aqueous environment while long nanofiber in nonpolar toluene can be ascribed to the different hydrogen bonding and polarity in various solvents 86. For example, Yan et al. validated that solvents capable of forming hydrogen bond, including ethanol, DMF, and acetone, can expedite the nanofiber formation of diphenylalanine 87. Their groups also summarized that the cyclic dipeptides possessed excellent hydrogen bonding-forming ability, which exhibited great value in drug delivery and cancer therapy. Besides, the hydrogen-bond between hydrogen atoms and electronegative atoms in peptides and drugs could stabilize the formed nanostructures. For instance, by integrating cyanuric acid and peptide, Zhang et al. designed a new amphiphilic peptide (CA-C11-GGGRGDS). Once mixing this peptide with methotrexate (MTX), the complementary hydrogen bonding interaction between cyanuric acid in peptide and MTX would facilitate the increase of hydrophobicity of the system, thus leading to peptide/MTX nanostructures 88. Yang et al. employed the co-assembled strategy to fabricated doxorubicin-loaded gel, both the hydrogen bond and aromatic π-π stacking played the critical role in the co-assembly process 89.

### 3.5 Van der Waals force

Van der Waals force belongs to a short-range force without directivity and selectivity. Since the strength of van der Waals forces is weaker than that of hydrogen bonds, it rarely acts as the main force to promote peptide self-assembly, but always cooperates with other interactions as a driver. Wang and colleages utilized steered molecular dynamics (MD) simulations and umbrella sampling techniques to investigate the formation mechanism of KIIIIK in their study 90. The findings revealed that the formation of the intra-sheet structure was influenced by both van der Waals and electrostatic interactions, whereas the inter-sheet structure was mainly governed by the van der Waals force. Van der Waals forces are also a crucial force to regulate the co-assembly process between peptides and drugs. Li et al. designed a hybrid supramolecular hydrogel (Lev/Dex-SA-RGD) through the co-assembly of dexamethasone-peptide amphiphile (Dex-SA-RGD) and antibiotic levofloxacin (Lev) 91. Further study indicated that the balance of electrostatic forces and Van der Waals forces play an important role in the process of co-assembly.

In general, these weak interactions were always prone to be influenced by the surrounding environment, such as pH, temperature, solvent, and stimuli. In the co-assembly process, it is always necessary to rely on the cooperation of multiple interactions to produce multifunctional and stable nanostructures. The research group represented by Yan et al. has constructed a variety of structurally stable supramolecular delivery systems through the cooperation of a variety of non-covalent forces between peptides and drugs 92. Overall, the non-covalent forces play an indispensable role in the co-assembly of peptides and drugs, which would be significantly considered when designing co-assembled nanostructures for drug delivery.

## 4. Co-assembly of peptide and drug molecules for cancer therapy

In the early stage of cancer, surgical treatment is the main treatment for low-risk patients. However, in the middle and late stages, systematic treatment is of significant importance. Systematic treatment of cancer includes chemotherapy, phototherapy, gene therapy, radiotherapy, immunotherapy, and combination therapy. In order to achieve the optimum therapeutic effects, carrier-based delivery is usually needed. Thanks to the good biocompatibility, tunable structures and excellent biodegradability of peptides, the peptide assemblies-based delivery system exhibits broad application prospects in drug delivery.

Great progress has been made in the delivery of anticancer molecules with peptide assembly strategies, which is attributed to good biocompatibility, enhanced accumulation in tumor sites, and cell permeability. Traditional encapsulation is always troubled by drug leakage, affecting efficacy. Encouragingly, the co-assembly delivery system formed by the interaction between peptide and drug molecules has been approved to conquer the above problems. The co-assembly strategy exhibits the following advantages: (Ⅰ) Enhanced stability of therapeutic agents *in vivo*; (Ⅱ) Controlled release profile of therapeutic agents; (Ⅲ) Improved drug loading efficiency; (Ⅳ) Enhanced targeting capability; (Ⅴ) Excellent therapeutic efficacy. The following focused on the recent research about the co-assembly of peptides with various drug molecules (Table 1).

### 4.1 Co-assembly of chemotherapeutic agents and peptide

#### 4.1.1. Chemotherapeutic agents-peptide co-assembly for chemotherapy

Chemotherapy lays a foundation for traditional cancer treatment. Chemotherapeutic drugs exert their anti-cancer efficacy through destroying the cell cycle process. According to the mechanism of curative effects, chemotherapeutic drugs can be classified into five categories, including alkylating agent, antimetabolic agent, tubulin-binding drug, topoisomerase inhibitor, and antineoplastic antibiotics 93-97. Alkylating agent includes nitrogen mustards, platinum drugs, and oxazsaphosphorines, which always destroy cancer cells by covalently binding with the DNA and RNA of cancer cells. With a similar structure to metabolites *in vivo*, antimetabolic agents exhibit their efficacy by specific antagonism with the essential metabolite*.* Pyrimidine drugs, purine drugs, and antifolates are commonly used as antimetabolic drugs. Taxanes and vinca alkaloids belong to tubulin-binding drugs, which can inhibit the formation of the microtubule by binding to tubulin and further kill cancer cells. Topoisomerase inhibitors can significantly inhibit topoisomerase activity and promote the breakage of DNA strand breakage. Antineoplastic antibiotics kill cancer cells by inserting into DNA at particular sites to further destroy DNA strands. However, there are still some major limitations in the application of chemotherapeutic drugs. Drug resistance is a major contributor to the failure of chemotherapy, wherein drug efflux, gene mutations, enhanced DNA repair capability, and increased anti-apoptotic capability are often attributed as causes. Besides, chemotherapy also associates with serious side effects, poor targeting ability, and limited therapeutical efficacy.

However, the co-assembly of peptides in chemotherapeutic agents displays promising potential for addressing the issues of poor targeting and toxic side effects encountered with chemotherapy. The co-assembly strategy can exert a significant influence on the physicochemical properties and targeting ability of the drug delivery system. On the one hand, the co-assembly of peptides and chemotherapeutic agents works to tune the micromorphology and the mechanical properties of the delivery system; on the other hand, this co-assembly strategy can enhance the targeting ability and anti-tumor efficacy of chemotherapeutic agents.

Firstly, the micromorphology of nanostructures can be tuned by utilizing the co-assembled strategy, which will meet the different requirements of the delivery system. For example, He and his colleagues designed a cystitis-bridged peptide (ATKTA-S-S-ATKTA, abbreviated CBP), which could co-assemble with hydrophobic antitumor drug curcumin (CCM) to form glutathione (GSH)-responsive CCM-loaded micelles (CCM-CBP). The aromatic groups of curcumin interacted with the hydrophobic moiety of the peptide and formed new intermolecular hydrogen bonds, thus leading to microscopic morphological transformation from fiber to micelle. Additional findings revealed that CCM-CBP micelles displayed improved cellular uptake by cells and effectiveness against tumors 98. Zhong et al. designed Naphthylacetic acid-conjugated peptides (Nap-1), which consisted of self-assembled sequences, RGD residues, and histidine residues. Nap-1 could co-assemble with DOX·HCl through electrostatic interactions to form nanofiber for controllable drug release and efficient inhibition of cancer cells 85. Zhao et al. described CAP-NPs, which were formed by the co-assembly of a FAP-a responsive amphiphilic peptide (Ac-ATK(C18)DATGPAK(C18)TA, CAP), and Dox. After the co-incubation of CAP with Dox, the fiber structure was gradually transformed into stable nanospheres under the hydrophobic driving force, displaying the indispensable role of the hydrophobic drug in the process of co-assembly 99.

Secondly, the co-assembly strategy helps drugs target tumor cells or subcellular organelles and prolongs drug residence time at tumor sites, thus leading to enhanced therapeutical efficacy. Yang and his colleagues prepared Fmoc-FK (FK) and Fmoc-FKK (FKK), which could co-assemble with Dox in different ratios to obtain two kinds of peptide-Dox nanoparticles. These nanoparticles demonstrated excellent uptake of tumor cells due to the uniform particle size distribution (50-100 nm) and positive charge in the nanoparticles 100. Significantly, introducing targeting groups into the peptide structure for tumor overexpression receptors can enhance the targeting performance of the drug delivery system. Zhang et al. designed a novel amphiphilic cyanuric acid-peptide conjugate, which could form nanorods or nanofibers with different concentrations of methotrexate (MTX) under the interaction of hydrogen bonds between cyanuric acid in peptide and MTX. This MTX-loaded nanostructure showed superior anti-tumor effects compared with MTX, and the effects could be ascribed to the excellent targeting ability of the amphiphilic peptide 88.

#### 4.1.2. Chemotherapeutic agents-peptide co-assembly for combined chemotherapy

Combination chemotherapy depends on different chemotherapeutic drugs at a time to treat cancer, and by affecting the cell repair of cancer cells or different stages of the reproductive cycle, the efficacy of chemotherapy can be enhanced. The application of combination chemotherapy is guided by several general principles: 1) The drugs should be dosed appropriately to avoid superimposed toxicity**;** 2) They should exhibit different mechanisms and minimal cross-resistance; 3) Synergistic therapeutic effects should be observed at optimal ratios of drug doses; and 4) The drugs should have similar solubility to ensure efficient delivery to cells concurrently.

Peptide-based co-assembly offers a promising strategy to integrate multiple chemotherapeutic agents into a single nanosystem, enhancing the therapeutic effects and reducing adverse effects. Zhong et al. aimed to maximize the effectiveness of chemotherapy, designed an indomethacin (IDM)-peptide conjugate (IDM-GFFYGRGDH, denoted as IDM-1) for boosting the therapeutic efficacy of chemotherapeutics Dox. IDM-1 and Dox were co-assembled to produce IDM-1/DOX hydrogels, which had lower minimum gelation concentrations (MGCs) (1.5 wt%) than IDM-1 (2.0 wt%). The decrease of MGCs of IDM-1/DOX hydrogels indicated the Dox played a bigger part in assembly process. Further results showed that IDM-1/DOX hydrogels could continuously release Dox to synergistically inhibit A549 cancer cells 101. Recent research suggested that tyroservatide (YSV) exhibited antitumor efficacies despite the disadvantage of large dosages. In the context of the study, their group described a YSV moiety-containing octapeptides (FKFEYYSV, abbreviated as 1-YSV). Intriguingly, 1-YSV demonstrated a stable co-assembly with hydroxycamptothecin (HCPT), which could confer 1-YSV/HCPT hydrogel with excellent mechanical properties and enhanced therapeutic efficacy 102.

**Figure 5:**
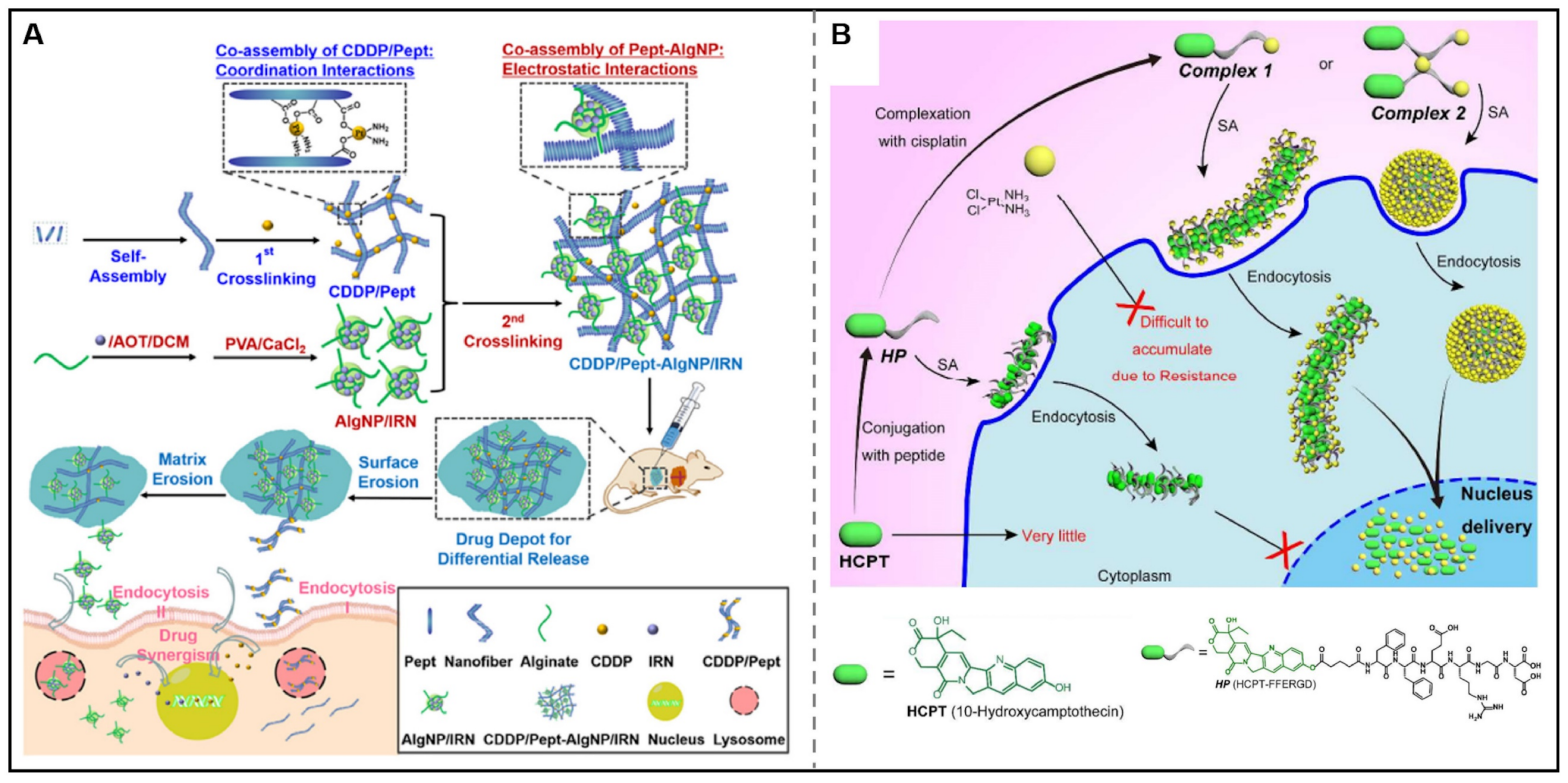
Chemotherapeutic agents-peptide co-assembly for combined chemotherapy. (A) Schematic illustration of the co-assembly of CDDP/Pept-AlgNP/IRN nanocomposite hydrogel loaded with dual drug cisplatin (CDDP) and irinotecan (IRN) for combination therapy. Adapted with permission from 103, copyright 2020, Elsevier Ltd. (B) Schematic illustration of supramolecular nanostructures for nuclear delivery of 10-hydroxycamptothecine (HCPT) and cisplatin (CDDP) against cancer cells. Adapted with permission from 104, copyright 2017, American Chemical Society.

Focusing on the temporal control in combined chemotherapy, the Zhong group also showed a nanocomposite hydrogel for the differential release of two chemotherapeutic drugs. In this design, a hydrogelator (Pept) was assembled with cisplatin *via* coordination interactions to form CDDP/Pept matrix. Additionally, emulsion cross-link technique was utilized to prepare nanoparticles loaded with irinotecan (IRN). Through a co-assembling process, CDDP/Pept-AlgNP/IRN nanocomposite hydrogel was obtained through the electrostatic interactions between CDDP/Pept and AlgNP/IRN. Notably, this nanocomposite hydrogel could be served as a platform for the tunable release of CDDP and IRN by tailoring the fraction ratios of AlgNP/IRN in the hydrogel, showing superior synergistic antitumor effects *in vitro* and *in vivo* (Figure 5A) 103. However, only delivering the drugs into the tumor cells couldn't yield optimal therapeutic effects as the action sites of some drugs were in the intracellular subcellular organelles. Yang's research group has made great progress in the utilization of co-assembly to improve the subcellular targeting ability of chemotherapeutic agents. They designed and synthesized HCPT-peptide conjugate (HCPT-FFERGD, abbreviated as HP). HP could interact with different equivalents of cisplatin and self-assemble into distinct nanostructures with different micromorphology (Figure 5B). The assembled nanostructure could be served as a “Trojan horse” carrier that could deliver dual anticancer drugs to reach the nucleus to efficiently kill tumor cells 104. After that, their research group employed the strategy of co-assembly for the nuclear delivery of rhein and cisplatin. They synthesized rhein-peptide conjugate (Rh-GFFYERGD and Rh-GFFYERGE), which could co-assemble with cisplatin to form Rh-gel. *In vitro* drug release experiments indicated that the cumulative release of cisplatinum was 34.9, 31.2, and 23.9% within 16 h from Rh-gel formed by incorporation of 4, 6, and 8 eq. cisplatinum, respectively. Rh-gel not only exhibited excellent nuclear accumulation properties but also displayed superior anti-tumor efficacy *in vitro* and *in vivo* 105. We envision co-assembly strategy can be further used in the clinic to improve the therapeutic effects of chemotherapeutic agents for combined chemotherapy.

Even though the stability and targeting ability of chemotherapeutic drugs have been greatly improved through co-assembly strategy, the effectiveness of chemotherapy is still unsatisfactory for some cancers. According to specific tumor types to design tumor microenvironment responsive co-assembled peptide delivery system may be an effective strategy to enhance curative effects and reduce side effects of chemotherapeutic drugs.

### 4.2 Co-assembly of radiosensitizer and peptide

#### 4.2.1. Radiosensitizer-peptide co-assembly for radiotherapy

Radiotherapy is one of the main methods to treat cancer, and its cure rate is about 50%. For patients with laryngocarcinoma, non-small cell lung cancer, gliomas or sarcomas, radiotherapy exhibits significant therapeutic effects and the survival rate is higher than that of other therapies. However, radiotherapy has been found to have unsatisfactory results in treating sarcoma, gastric cancer, and other types of cancer. Ionizing radiation, which is produced by X-ray, electron, proton, or other particle beam is greatly related to the effectiveness of radiotherapy. Reactive oxygen species produced by ionizing radiation can destroy DNA strands and inhibit the cell cycle, leading to tumor cell death 106, 107. Besides, endogenous danger signals can promote the release of cytokines and further boost the presentation of tumor-associated antigens, inducing the diversity of host T cells. Although the superiority of these novel radiotherapy strategies in tumor therapy, they can still cause various side effects, such as the occurrence of secondary tumors, radiation-induced pneumonia, lymphedema, and spinal cord injury 108-110.

Recently, nanomaterials have gained favorable achievements in enhancing radiotherapy sensitivity and reducing side effects. Among them, peptide assemblies have gained encouraging results in sensitive radiotherapy. For example, Ding et al. designed a curcumin-peptide conjugate, which could self-assemble to form Cur-SNF nanofiber. The nanofiber showed a superb performance in amplifying the radiosensitivity of cancer cells to ionizing radiation 111. Liu and other groups also reported impressive results of peptide assemblies in sensitizing radiotherapy 112, 113. The peptide-based co-assembly approach also provided an available strategy for improving the radiotherapy effects. Liu and coworkers developed a novel supramolecular hydrogel combining naproxen (Npx)-peptide conjugate (Npx-FFEY) and radiosensitizer cisplatin. The co-assembled supramolecular hydrogel exhibited enhanced radiosensitization effects by promoting the formation of Pt-DNA adducts and inhibiting the activity of COX-2 114.

#### 4.2.2. Radiosensitizer-peptide co-assembly for combined radiotherapy

Hypoxic tumor cells are prone to radiotherapy resistance. Radiotherapy combined with other therapies may induce promising outcomes. Recent research indicated that radiotherapy combined with PDT exhibits encouraging results, such as improving the quality of life of patients, reducing side effects, and enhancing the sensitivity of radiotherapy. ^125^I radioactive particles display huge advantages in radiotherapy due to their long duration time in tumors and negligible toxicity to normal tissue. Accordingly, Dai et al prepared an R_9_-based peptide derivative that integrated ^125^I-labeled cRGD and Ce6 to further co-assemble with miR-139-5p to afford nanoparticles (Ce6-R_9_-^125^I-RGD-MNPs) 115. The nanoparticles exhibited excellent targeted ability towards tumor cells and enhanced combined therapeutical efficacy between photodynamic therapy and radiotherapy.

**Figure 6:**
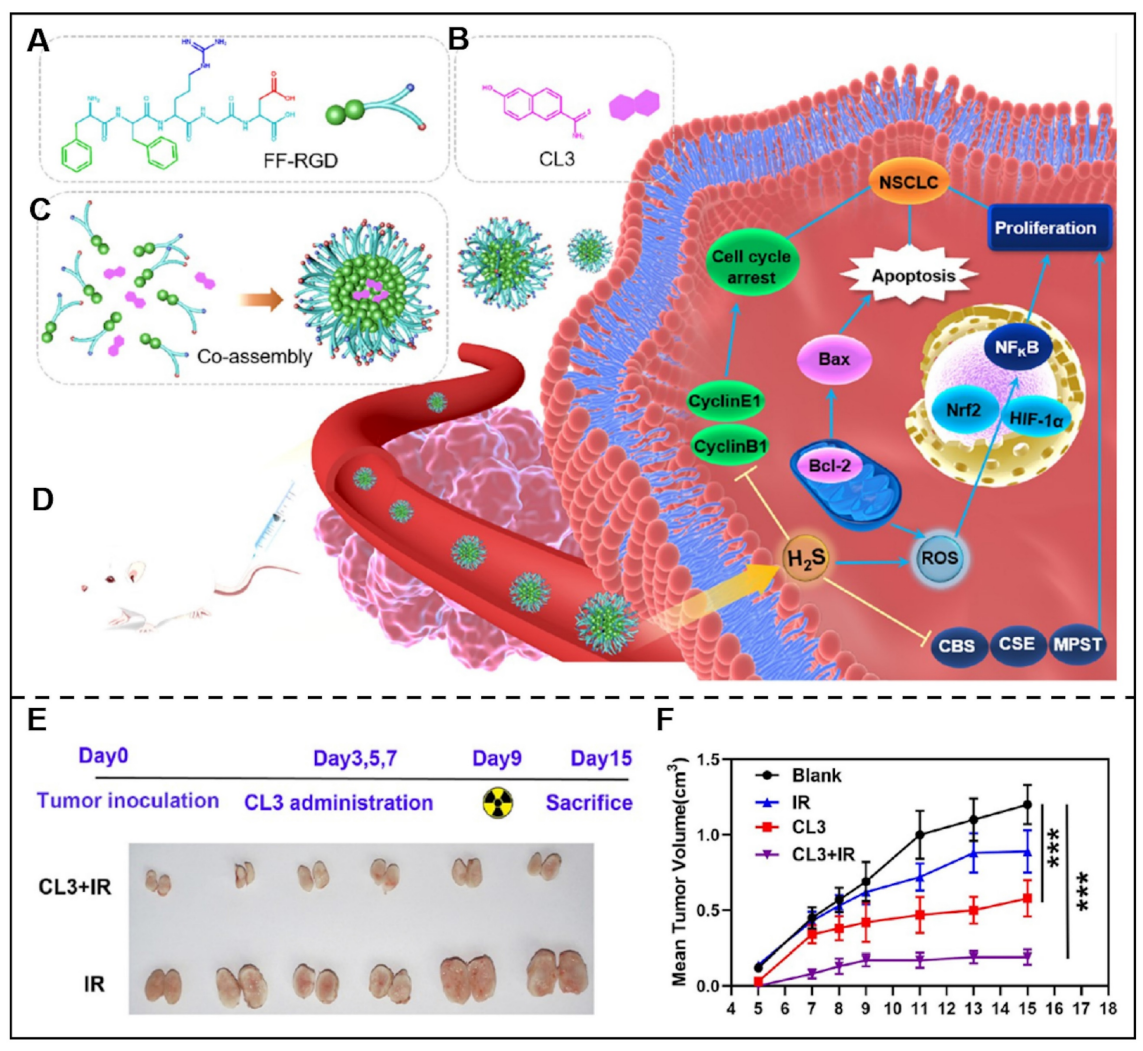
Peptide-based co-assembly for sensitized radiotherapy. (A) Chemical structure of FFRGD peptide. (B) Chemical structure of H_2_S Donor (CL3). (C) Schematic illustration of FFRGD peptide co-assembled with H_2_S into nanocarriers. (D) Schematic illustration of the co-assembled nanocarriers for cancer therapy. (E) Schematic illustration of representative xenografts were extracted from 2-Gy radiation (IR) or co-assembled nanocarriers+IR treated groups of nude mice. (F) Time curve of average tumor volume after nude mice were received with 2-Gy radiation (IR) or co-assembled nanocarriers+IR. Adapted with permission from 118, copyright 2022, American Chemical Society.

Radiotherapy combined with immunotherapy integrated efficient radiotherapy effects and immunological effects, displaying distinctive superiorities for tumor therapy. For example, Schoenfeld and his colleagues studied the efficacy of ipilimumab and 65 courses of radiation in 47 patients with consecutive metastatic melanoma and the results indicated a promising synergistic efficacy of this therapeutic regime 116. Inspired by these research findings, Gao et al. designed a novel peptide derivative NIA-D1, which integrated radio-sensitizer 2-(2-nitroimidazol-1-yl) acetic acid (NIA), matrix metalloproteinase-2 (MMP-2) responsive peptide sequence and PD-L1 antagonist. This peptide derivative co-assembled with dendritic cells (DCs) activators toll-like receptor (TLR) 7/8 ligand R848 and further formed NIA-D1@R848 nanoparticles. Once NIA-D1@R848 reached tumor sites, it would be decomposed under the overexpressed MMP-2 in the tumor microenvironment, and NIA-PLG, PD-L1 antagonist, and R848 were then released. The released NIA-PLG would promote the released antigen under radiation, and R848 boost the maturation of DC, while the PD-L1 antagonist would further alleviate T cell suppression, and they work together to induce cancer cell death 117. Tian et al. also constructed a novel co-assembled delivery system by integrating FFRGD and H_2_S donors (CL2/3) for effcienct combined chemo-radiotherapy (Figure 6) 118.

The integration of radiotherapy with co-assembly strategy have broken the limitations of radiotherapy, such as the reduced toxicity, enhanced therapeutical efficacy and extend the application range in combination. The efficacy of radiotherapy is heavily reliant on the quality of imaging technology employed. The combination of peptide-based co-assembly with advanced imaging technology may bring significant development for cancer treatment in further.

### 4.3 Co-assembly of gene therapeutic agents and peptide

#### 4.3.1. Gene therapeutic agents-peptide co-assembly for gene therapy

Gene therapy based on small interfering RNA (siRNA), plasmid DNA (pDNA) and RNA/DNA aptamers have become a burgeoning method for tumor therapy 119, 120. Among them, gene therapy represented by siRNA-based therapy has been widely studied thanks to the significant advantages of anti-tumor therapy. Although siRNA has limited protein targets, it can interfere with almost all gene transcripts. According to phase I clinical trials, siRNA can prevent various gene targets and lead to cancer cell death, which has been verified in patients with cancer 121. siRNA and other gene therapeutic agents are prone to degradation by enzymes within the body and exhibit instability in blood circulation. In addition, due to their larger size and negative electricity, it is difficult for gene therapeutic agents to cross cell membranes freely. Thus, it is necessary to find a suitable method to improve the *in vivo* stability and delivery efficiency of gene therapeutic agents.

**Figure 7:**
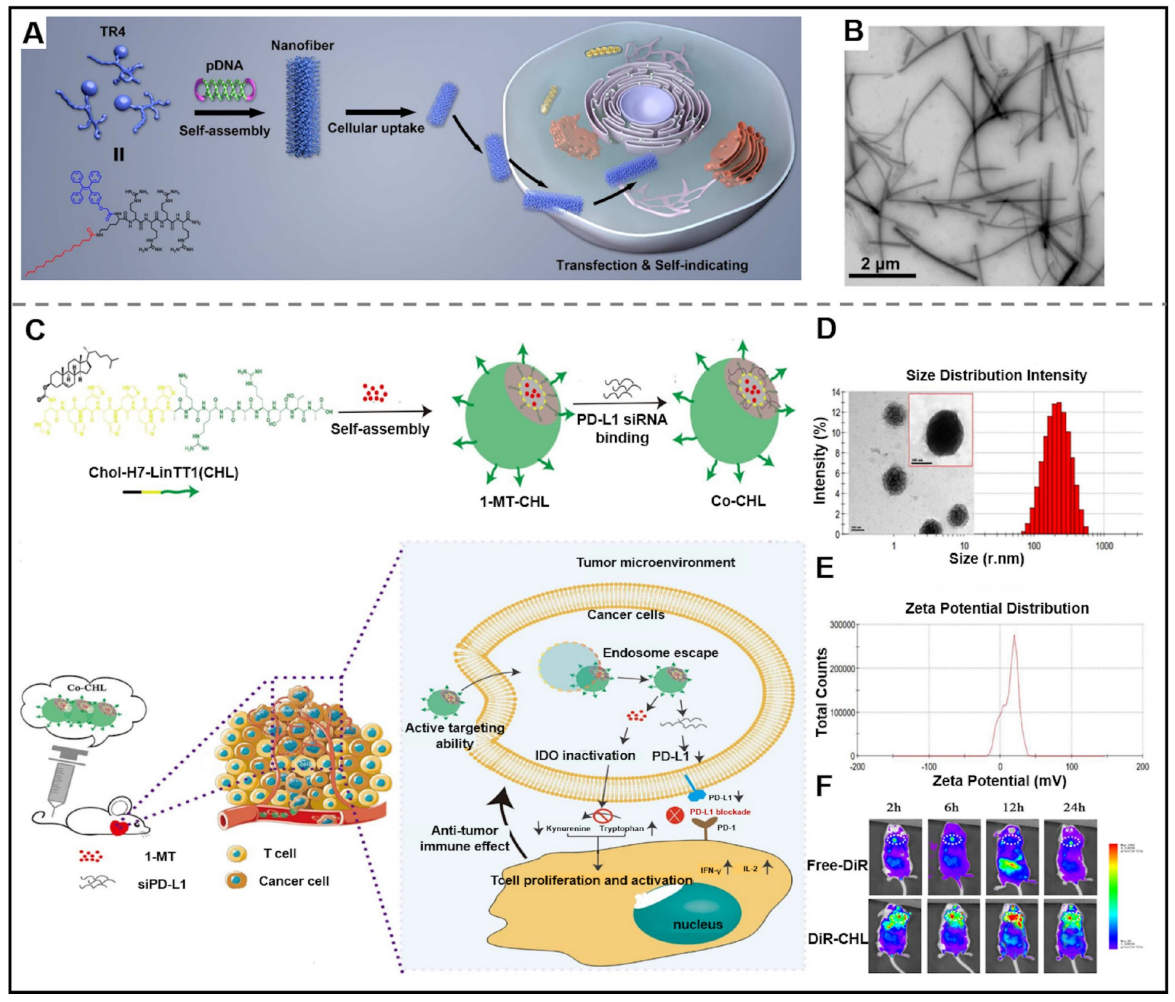
Gene therapeutic agents-peptide co-assembly for cancer therapy. (A) Schematic illustration of the co-assembled pDNA@TR4 nanofibers by using a short peptide derivative (TR4) and plasmid DNA (pDNA) for traceable gene delivery. (B) TEM image of pDNA@TR4 complexes, bar: 2 μm. Adapted with permission from 125, copyright 2017, American Chemical Society. (C) Schematic illustration of Co-CHL NPs, by the assembly of CHL (Chol-HHHHHHH-AKRGARSTA), siRNA of programmed cell death ligand 1 (siPD-L1) and 1-methyl-DL-tryptophan (1MT) and the mechanism of Co-CHL NPs for cancer treatment. (D) The size distribution characteristic of Co-CHL NPs. (E) Zeta potential of Co-CHL NPs. (F) *In vivo* images of 4T1 tumor-bearing mice after receiving tail vein injection of free DiR or DiR-loaded Co-CHL NPs in different times. Adapted with permission from 133, copyright 2019, American Chemical Society.

Recently, researchers have designed new delivery vectors to improve its shortcomings, bringing the utilization of gene therapeutic agents in clinical cancer treatment into reality 122. Supramolecular peptides are increasingly recognized as the ultimate delivery carrier due to their superior capacity to enhance the stability of gene therapeutic agents and promote their delivery efficiency, amongst all other delivery carriers. Peptides can be co-assembled with gene therapeutic agents to form nanostructures with diverse structures, such as micelles, nanoparticles, nanotubes, and nanofibers. The driving forces of co-assembly between peptides and gene therapeutic agents mainly include non-covalent interactions, such as hydrophobic interaction, hydrogen bonds, electrostatic interaction, and so on. It is noteworthy that the peptide used for efficiently delivering gene therapeutic agents needs meet specific structural requirements, which include (Ⅰ) a hydrophobic sequence for endowing peptide with self-assembling; (Ⅱ) a hydrophilic segment for balancing the amphiphilicity; (Ⅲ) a positively charged amino acid residues for expeditiously binding with siRNA and improving the loading stability of siRNA; (Ⅳ) targeted groups for improving targeting ability towards cancer cells. The utilization of co-assembly between peptide and gene therapeutic agents is a breakthrough in improving the *in vivo* stability of therapeutic agents. For instance, Deshayes et al. described a novel D-cell penetrating peptide (RICK), which was the retro-inverse transformation of L-parental CADY-K. RICK/siRNA assemblies were able to prevent the degradation of siRNA and inhibit gene expression 123. Ramezani et al. presented a nanoplatform consisting of peptides functionalized with aptamers. Firstly, an arginine-rich peptide (LWMP) was functionalized with KALA and a nuclear localization signal (NLS) to obtain a novel peptide KLN (KALA-2LWMP-NLS). And then, the KLN peptide was assembled with plasmid DNA (pDNA), and further modified with AS1411 aptamer (Apt) to form KLN/pDNA/aptamer nanoparticles. The results indicated that the developed nanoplatform exhibited excellent stability which could prevent pDNA from degradation. The nanoparticles also showed satisfactory targeting ability towards specific tumor cells and enhanced cellular uptake, thus demonstrating superior gene transfection 124. Adopting a similar strategy, Liang et al. synthesized a tetraphenylethene (TPE)-conjugated peptide TR4, which co-assembled with plasmid DNA (pDNA) into pDNA@TR4 nanofibers (Figure 7A-B). pDNA@TR4 nanofibers were stabilized by the noncovalent interactions between pDNA and TR4, thus leading to enhanced stability of gene/vector complexes 125.

In addition, the co-assembly of peptides and gene therapeutic agents can improve the delivery efficiency, boost gene transfection efficiency and improve therapeutic efficacy. For example, Li et al. described peptide Wpc, which was obtained by modifying the Met residues in Fmoc-RRMEHRMEW with reducible cross-linkers. Peptide Wpc could co-assemble with siRNA at the optimum peptide/siRNA ratio to form peptide-siRNA nanoparticles. These nanoparticles demonstrated improved cellular uptake of siRNA and elevated transfection effectiveness. The* in vivo* experimental results showed that peptide-siRNA nanoparticles could significantly inhibit tumor growth with low side effects 126. In addition, their group also designed a novel co-assembled Wpc/AS1411 nanocarrier for efficient delivery of aptamer AS1411 to enhance anticancer therapeutical efficacy 127. Among multifunctional peptides, cell-penetrating peptides, such as arginine-containing peptides, displayed great potential in siRNA delivery due to their excellent transmembrane ability. Mollenhauer and co-works construed a siRNA nanocomplex delivery system based on the respective co-assembly of two DMBT1-derived-peptides with siRNA. They synthesized a DMBT1-derived peptide (SRCRP2-11, GRVEVLYRGSW) to bind with tdTomato1 siRNA. In addition, SRCRP2-11-R was synthesized by replacing the glutamic acid (E) at position 4 of SRCRP2-11 with an arginine. By changing the ratios of peptide and siRNA, the size of two peptide-siRNA complexes could be tuned and the two peptide-siRNA complexes exhibited efficient gene silencing in MCF7-recombinant cells 128. Inspired by that superior binding affinity with cytomembrane, Chen et al described a novel arginine-rich peptide NP1 (STR-H_16_R_8_) to aid siRNA delivery. Their results indicated that NP1 could co-assemble with siRNA into nanoparticles (<200 nm), which displayed improved siRNA knockdown efficiency in 3D spheroids of colon cancer cells 129. To enhance the cellular uptake of siRNA, their group designed peptide/siRNA complexes by combining STR-HK peptides with siRNA, which apparently enhanced the inhibitory effects on tumor proliferation 130. Xia et al. designed a multifunctional peptide-conjugated TDNCP, which contains the following parts: (Ⅰ) targeted peptide sequence (DGR or RGD); (Ⅱ) nuclear targeting sequence (KRRRR); (Ⅲ) cell-penetrating peptide (RRRR); (Ⅳ) an AIE molecule. TDNCP co-assembled with DNA oligonucleotide (ASO) into stable TNCP/ASO-NPs through electrostatic interaction. TNCP/ASO-NPs showed a strong inhibition on tumors and a great interference effect thanks to their outstanding targeting function 131.

#### 4.3.2. Gene therapeutic agents-peptide co-assembly for combination gene therapy

Current clinical research on single-agent therapy has displayed considerable therapeutical efficacy, but its side effects are nonnegligible. Multiple-agent therapy works to synergistically kill cancer cells *via* different action mechanisms. In terms of combination therapy in anti-tumor, there are several reports on gene therapeutic agents-based supramolecular peptide co-assembly in combined gene therapy. Xia et al. designed a peptide-conjugated AIEgen (FC-PyTPA), which co-assembled with siRNA into FCsiRNA-PyTPA through electrostatic interactions. *In vitro* and *in vivo* experiments demonstrated that the production of ROS, gene interference of C_siRNA_ and formation of nanofibers synergistically promote cancer cell death 132. Gao et al. designed a novel drug delivery system (Co-CHL NPs) based on cholesterol-peptide conjugate (Chol-HHHHHHH-AKRGARSTA, CHL) for efficient delivery PD-L1 small interfering RNA (siPD-L1) and 1-methyl-DL-tryptophan (1MT). Co-CHL NPs exhibited synergistic antitumor immune response thanks to the dual blockade of an immune checkpoint of siPD-L1 and 1MT (Figure 7C- F) 133.

Although supramolecular peptide assembly displays huge potential in the delivery of gene therapeutic agents, peptide assembly-mediated gene therapy has not been applied in clinical due to several important reasons. First, the designed co-assembly system should be stable and capable of resisting the degradation of enzymes in blood circulation. To this end, the driver of the co-assembly should not only include electrostatic forces, but also other non-covalent forces, such as hydrophobic interaction and hydrogen bonds. Second, multifunctional peptides should be designed to efficiently target and bind with different tumor cells to increase cellular internalization. Last but not least, the assembly should respond to the intracellular environment and precisely release siRNA or DNA in target sites. The co-assembly for gene therapy has gained some advances, which can be used for tumor therapy and gain satisfactory results as the development of structural simulation and modification and the transport mechanism of the peptide assemblies.

### 4.4 Co-assembly of phototherapeutic agents and peptide

#### 4.4.1. Photothermal conversion agents-peptide co-assembly for photothermal therapy

Phototherapy mainly includes photothermal therapy (PTT) and photodynamic therapy (PDT), which is a potential therapeutic method to enhance anti-tumor efficacy by utilizing photosensitizers and external laser irradiation. PTT is a new phototherapy method different from PDT in the treatment of tumors, which achieves superior anti-cancer efficiency by employing photothermal conversion agents (PTCAs) that generate vibrational energy (heat) when exposed to NIR light 134, 135. The heat generated by light could increase local temperature and induce local tumor tissue ablation without affecting normal tissues. Although some progress has been made in phototherapy, its clinical applications are still challenged by many problems, including certain thermal damage to normal skin tissue and limited therapeutic effects in PTT. In addition, the rapid clearance of photosensitizer greatly weakened its anticancer efficacy. In recent years, researchers have committed to looking for corresponding solutions to the drawbacks existing in phototherapy, and the following methods, such as enhancing the targeting ability, utilizing reduced-temperature PTT, using combination therapies, and designing intelligent responsive PTT were applied.

**Figure 8:**
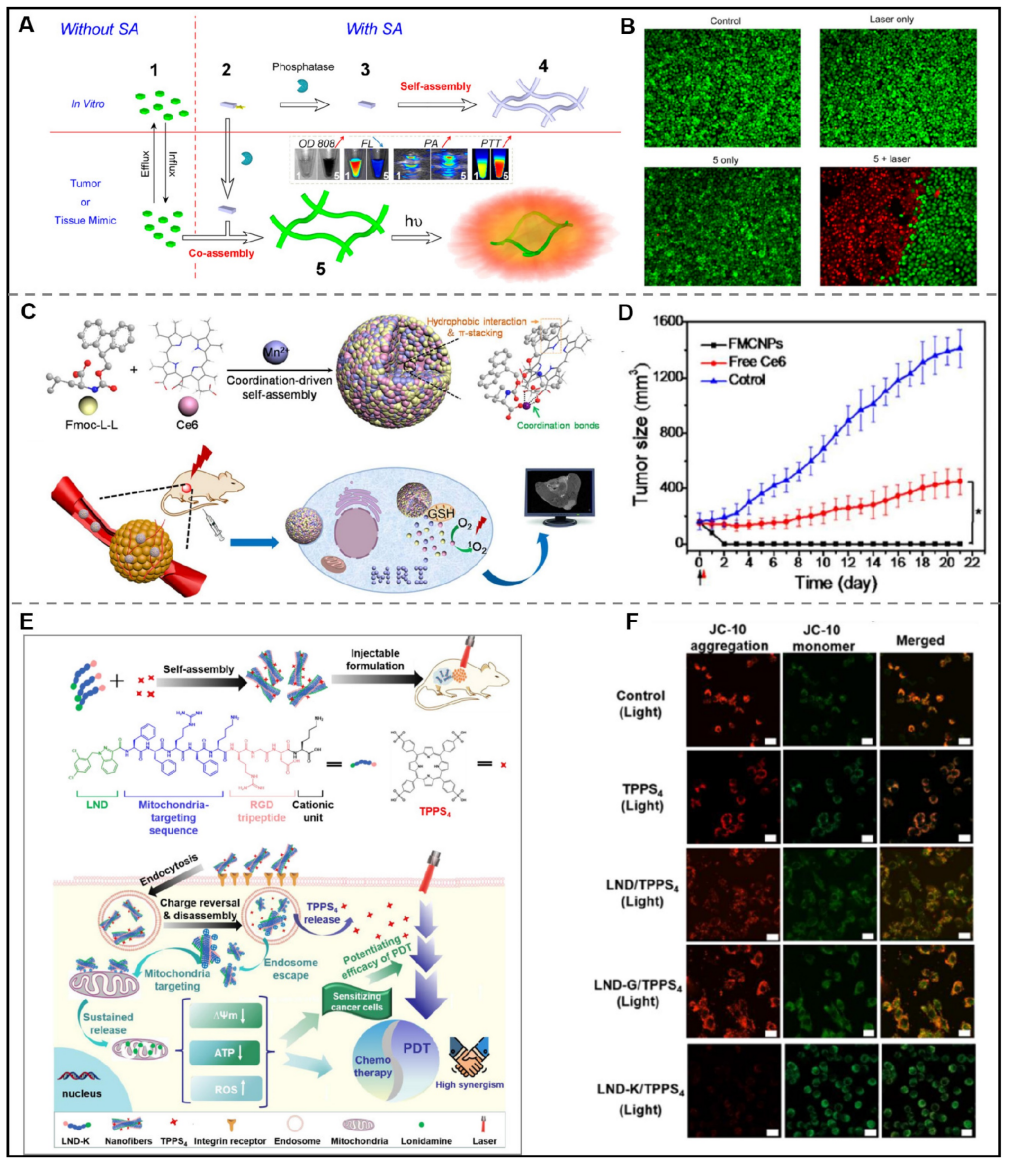
Photothermal conversion agents-peptide co-assembly for photothermal therapy. (A) Phosphatase-instructed *in situ* co-assembly of NapFFKYp (2) and indocyanine green (ICG, 1) to form nanofibers (5) for photothermal therapy (PTT). (B) Calcein AM/PI staining of HeLa cells after incubating with indocyanine green (ICG)-doped nanofibers with laser irradiation (808 nm, 1 W/cm^2^, 5 min). Adapted with permission from 136, copyright 2015, American Chemical Society. (C) Schematic illustration of the Fmoc-L-L/Mn^2+^/Ce6 nanoparticles (FMCNPs) which were formed by co-assembling of peptide derivative Fmoc-L-L, MRI contrast agent Mn^2+^ and photosensitizer Ce6 *via* the cooperation of multiple non-covalent interactions for MRI-guided PDT. (D) Time curve of average tumor volume of MCF7-tumor-bearing mice after receiving various groups treatments. Adapted with permission from 141, copyright 2018, American Chemical Society. (E) Schematic illustration of the supramolecular nanofibers, assembled by LND-peptide conjugate (LND-K) and photosensitizer TPPS_4_ for the efficient delivery of lonidamine (LND) and synergistic photodynamic therapy. (F) CLSM images of LND-K/TPPS4-treated cells with JC-10 for indicating mitochondrial membrane potential. Red, JC-10 aggregates; Green, JC-10 monomer. Adapted with permission from 147, copyright 2023, Elsevier Ltd.

Many nanomaterials have been exploited for efficiently delivering PTCAs. Among them, the co-assembly of peptides and PTCAs demonstrates higher potential. *In situ* assembly triggered by over-expressed enzymes, GSH, ions, etc in tumor microenvironment has been a promising approach to cancer treatment. Chen and his colleagues investigated the co-assembly of ICG and NapFFKYp triggered by enzymes. In the presence of over-expressed alkaline phosphatase (ALP) in the tumor microenvironment, NapFFKYp could convert into NapFFKY. And then, NapFFKY co-assembled with ICG into nanofibers. Compared with free ICG, ICG nanofibers showed partial fluorescence signal quenching, augmented photothermal and photoacoustic signal intensity, and increased cellular uptake (Figure 8A-B) 136. In addition to* in situ* assembly, *in vitro* assembly is another typical method for co-assembling peptide and photosensitizers. Yan et al. adapted biliverdin (BV), endogenic NIR-absorbing pigments, to construct a photothermal nanomaterial to enhance the efficiency of tumor diagnosis and treatment. They employed BV, histidine-containing peptide (Z-Histidine-Obzl, abbreviated as ZHO), and Mn^2+^ to fabricate ZHO/BV/Mn^2+^ nanoparticles (ZBMnNPs). ZHO was selected as an assembly agent that could coordinate with Mn^2+^ through the imidazole residue and co-assemble with BV under the aromatic-aromatic interaction. The result was impressive that, ZBMnNPs not only demonstrated selectivity towards tumors with efficient photothermal treatment effects but also displayed excellent photoacoustic and magnetic resonance imaging 137.

#### 4.4.2. Photodynamic sensitizers-peptide co-assembly for photodynamic therapy

PDT is an effective anti-cancer method, which exerts anti-cancer efficacy with the production of ROS by utilizing photosensitizer and external laser irradiation. There are two key parts to this process, one is that the photosensitizer can effectively enter the action site; another one is that the photosensitizer is activated by external laser irradiation 138, 139. The production of ROS in PDT can lead to cell death. Besides, PDT could also block tumor blood vessels and deprive the nutrition of tumor tissue, resulting in cancer cell death. In addition, PDT presents considerable impacts on the immune system, which inhibit tumor cell proliferation through immunostimulation or immunosuppression.

However, the hydrophobicity and non-selective distribution of photosensitizers lead to their aggregation and limited therapeutic effects, which hinders their clinical usage. The peptide-mediated assembly provides a new strategy to solve these problems. Notably, the benzene rings and hydrophobic residues of photosensitive molecules enable it to form a non-covalent force with peptide molecules and further promote the assembly process of peptides. In this context, Bai et al. employed a one-step co-assembly method to construct peptide-based nanoparticles for two-photon PDT. Here, the authors designed a peptide derivative Fmoc-L_3_-OMe which could co-assemble with m-THPP to form porphine doped peptide-based nanoparticles under the synergistic effect of aromatics and hydrophobicity interactions. Further results revealed that the nanoparticles could exert high ^1^O_2_ productivity and tumor cell inhibition effects* in vitro* under two-photon irradiation 140.

In order to exert optimum PDT efficacy, it is essential for photosensitizers to play their roles to reach tumor sites *via* passive or active targeting. However, the photosensitizers may be degraded in the systemic circulation due to the complex components in the body fluid, which will hinder the application of nanodrugs for PDT. Metal ion-mediated coordinated assembly may be an effective way to tackle this problem due to the stabilization of nanodrugs and their important role in biological regulation. Recently, Yan et al. developed two peptide derivatives, diphenylalanine (H-F-F-NH_2_·HCl, CDP) and Fmoc-L-Lys, respectively. When mixed chlorine6 (Ce6) with CDP or Fmoc-L-Lys, uniform spherical CDP/Ce6 nanoparticles or Fmoc-L-Lys/Ce6 nanoparticles would form. The structural stability of the obtained nanoparticles relied on the hydrophobic and π-π interactions between peptide and Ce6. Remarkably, the nanoparticles showed satisfactory PDT efficacy *in vitro* and *in vivo* 92. A similar effect can be seen in the co-assembly of amphiphilic peptide (9-fluorenylmethyloxycarbonyl-L-Leucine, Fmoc-L-L), MRI contrast agent (Mn^2+^), and photosensitizer (Ce6). Yan and his colleagues created Fmoc-L-L/Mn^2+^/Ce6 nanoparticles (FMCNPs) by co-assembling Fmoc-L-L, Mn^2+^, and Ce6. These nanoparticles are more stable due to the involvement of π-π, coordination, and hydrophobic interactions. Under the experimental conditions, FMCNPs exhibited significant tumor eradication in mice. In addition, the anti-tumor efficacy could be monitored by utilizing MRI in the presence of Mn^2+^ (Figure 8C-D) 141. Employing the similar strategy, their group described a novel nanodrug integrating photosensitizer, Zn^2+^, and metal-binding peptide to improve the antitumor PDT 142.

Recent advances demonstrated that tumor hypoxia was one of the primary factors hindering the efficacy of PDT 143. To tackle this problem, Sun et al. designed an arginine-conjugated peptide derivative 9-fluorenylmethyloxycarbonyl-Leu-Leu-Leu-Arg-OH (Fmoc-L_3_-Arg). The Arg segment in Fmoc-L_3_-Arg could be oxidized to NO and further combat the tumor hypoxia. In addition, Fmoc-L_3_-Arg could co-assemble with 5,10,15,20-Tetrakis (4-hydroxyphenyl) porphyrin (THPP) to form spherical THPP/Fomc-L_3_-Arg nanoparticles, which could not only rapidly release THPP in the tumor microenvironment and produce ROS, but also produce NO and inhibit mitochondrial function, thus leading to enhanced PDT efficacy 144.

#### 4.4.3. Phototherapeutic agents-peptide co-assembly for combined phototherapy

Phototherapy combined with other treatments may reduce the dosage of therapeutic agents, reverse drug resistance and produce synergistic antitumor effects, which always depned on the co-administration of photosensitizers and other therapeutic drugs. Photosensitizers can induce the generation of heat or ROS under NIR light and then induce the destruction of the tumor, exhibiting great potential in the application of phototherapy-combination therapy. Wang et al. have verified that photosensitizers IR780-instructed tumor therapy could be improved by the chemotherapeutic drug doxorubicin (DOX). They constructed a nucleus-targeting nanoparticle system through the assembly of YGRKKRRQRRRC-IR780 (TAT-IR780) and Dox for synergistic phototherapy and chemotherapy 145. Besides, phototherapy combined with immunotherapy demonstrates an potential solution to prevent tumor cell metastasis and recurrence, which can be ascribed to the amplified immune response or reduced immunosuppressive effects of the two combinations. Yan et al. created supramolecular TP5-ICG nanofibrils by utilizing the co-assembly of immunomodulatory thymopentin (TP5, RKDVY)) and indocyanine green (ICG) for phototherapy immunotherapy of pancreatic tumors. The treatment was able to induce the proliferation of antitumor immune cells, exerting excellent effects of inhibiting tumor growth and metastasis 146. Sensitizing cancer cells is a feasible method to reduce the toxic side effects and enhance the efficacy of photosensitizers. Considering this, Zhong et al. designed a lonidamine (LND)-conjugated peptide (LND-K) that efficiently targets the mitochondria of cancer cells. LND-K could co-assemble with photosensitizer TPPS_4_ into LND-K/TPPS_4_ nanofibers through electrostatic interactions. Thanks to the superior targeting ability of peptide, LND could be supplied progressively to both cancer cells and mitochondrial sites, which allowed it to synergistically kill cancer cells by inducing mitochondrial dysfunctions (Figure 8E-F) 147. Targeted delivery of chemotherapeutics affords a feasible strategy for enhancing the phototherapeutic effects. Generally speaking, combined phototherapy is a desirable way to improve anti-tumor efficacy, which will certainly gain a notable place in the clinical treatment of cancer in the future.

At present, the penetration depth of light limits the phototherapy to superficial tumor, such as melanoma and breast cancer. With the development of novel photosensitizers in the far infrared region, advanced fiber optic technology and endoscopic technology, peptide-based phototherapy is gradually being applied to deep diseases, which will break the traditional depth limitations and make greater progress in various cancer therapy.

### 4.5 Co-assembly of immunotherapeutic agents and peptide

#### 4.5.1. Immunotherapeutic agents-peptide co-assembly for immunotherapy

Immunotherapy is a very promising mean during cancer therapy, which exerts anti-cancer efficacy with the help of immune cells in host lymphatic tissue and anti-tumor immune cells in the tumor microenvironment. The anti-tumor immune reaction induced by immunotherapy can not only boost systemic immune monitoring, and destroy primary and disseminated tumors, but also build prolonged immune memories to prevent carcinoma recurrence. With relentless efforts, immunotherapy strategies such as immune checkpoint inhibitors-based therapy and chimeric antigen receptor (CAR) T cell therapy has been used in the clinic 148. In addition, a considerable number of immunotherapies have entered the preclinical or clinical trials stage. Most immunotherapies are classified into the following categories: immune checkpoint inhibitor, engineered T cell therapy, lymphocyte activator, vaccine, bispecific antibody, and oncolytic virus 149-152. Although immunotherapy has gained impressive results in some patients, it is still hindered by some problems in clinical usage. For example, due to the acquired resistance after immunotherapy, immune response rate efficiency is dissatisfactory, which hinders its clinical application.

Recently, considerable advancements in adjusting immune response and enhancing immunotherapeutic effects have been found in peptide-based assembly, which could be used as an antigen, delivery carrier, immunomodulator, or vaccine adjuvant for mono immunotherapy and combinatorial immunotherapy to treat tumors. Peptides are composed of amino acids, which are potential antigens for immunotherapy. Antigen peptides mainly include immunogenic and immune reactive peptides. Immunogenic peptides can induce an immune response, while immune reactive peptides can bind to substances to induce its immune response. Recent studies have shown that some antigenic peptides such as Q11 (Ac-QQKFQFQFEQQ-Am), have great potential in clinical anti-tumor treatment 153. However, single molecule antigenic peptides are easy to be rapidly degraded *in vivo*, leading to poor antitumor immunity. On the basis of the supramolecular co-assembly technology, the assembled antigen peptide conquers the deficiency of single molecule antigen peptide, and the superiority of assembled antigen peptide includes the following aspects: (Ⅰ) Diversified structure after specific modification; (Ⅱ) Enhanced tumor accumulation; (Ⅲ) Increased stability and extended retention time *in vivo*; (Ⅳ) Reinforced immunogenicity and immunoreactivity. For example, Sheng et al. designed a novel cationic polypeptide (10 K-Adpgk) by modifying neoantigen peptide (Adpgk) with 10 lysines. 10 K-Adpgk was then co-assembled with CpG oligodeoxynucleotides (CpG ODN) adjuvant into nanocomplex (PCNPs) for delivering neoantigen and adjuvant. The results showed that PCNPs not only displayed a preferable capability to prompt antigen-presenting process but also boosted the activity of cytotoxic T cells, demonstrating impressive suppression of colorectal tumors 154. Similarly, Wang and co-workers reported a nanocomplex vaccine for cancer immunotherapy. The nanocomplex vaccine was prepared by the co-assembly of the cationic peptide-conjugated epitope (Epitope-R_8_) and toll-like receptor 9 (TLR9) agonist CpG. The driving force of co-assembly relied on the electrostatic interactions between the oppositely charged peptide-conjugated epitope and CpG. Further results indicated that this nanocomplex vaccine could not only enhance the immunogenicity of peptides and elicit strong cytotoxic CD8^+^ T-cell immune responses, but also exhibit synergistic antitumor effects with immune checkpoint inhibitors 155.

**Figure 9:**
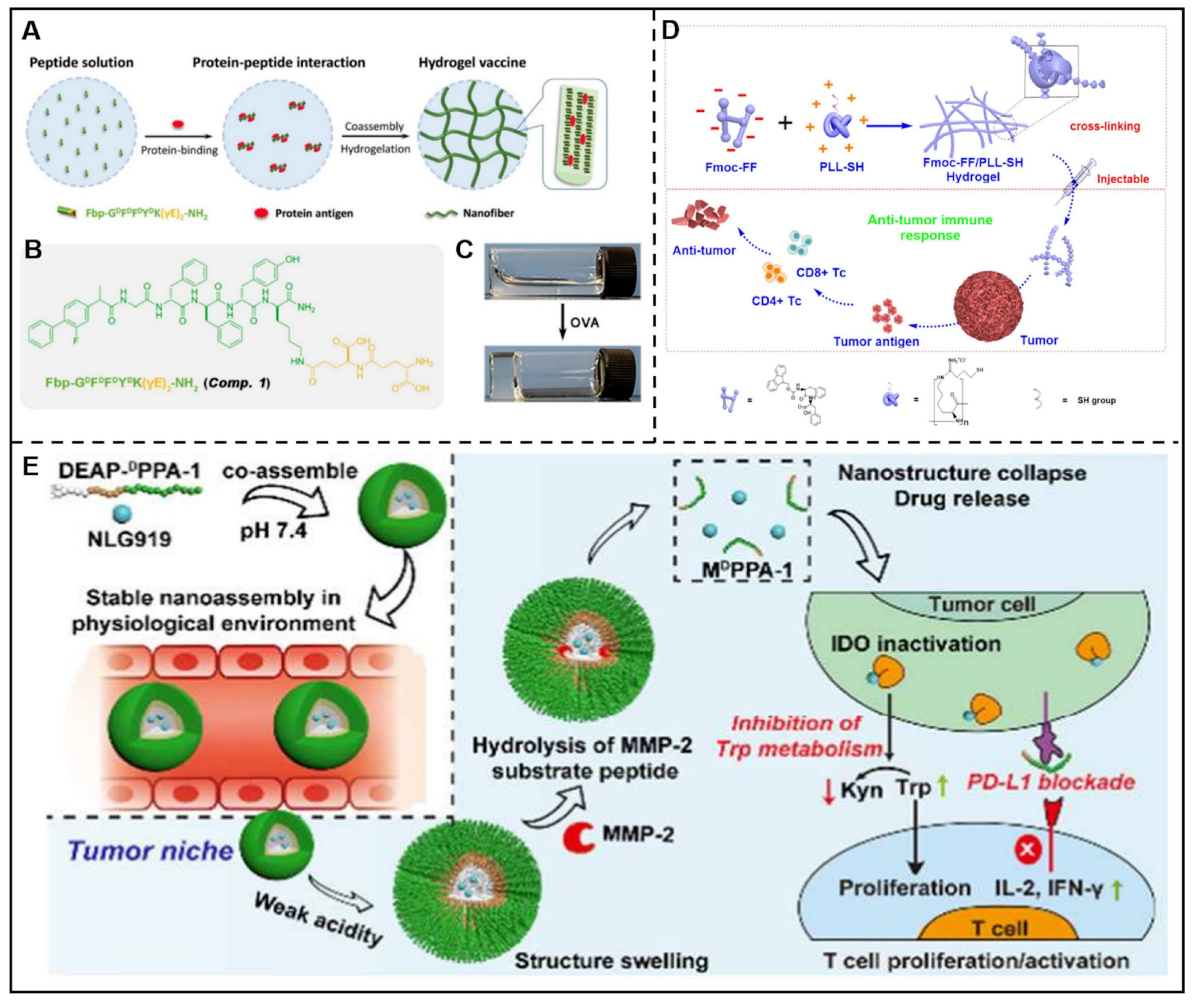
Immunotherapeutic agents-peptide co-assembly for immunotherapy and combined immunotherapy. (A) Schematic illustration of the preparation of OVA-induced co-assembled hydrogel vaccine. (B) Chemical structure of Comp.1. (C) Optical images of co-assembled hydrogel vaccine after addition OVA into Comp.1 solution. Adapted with permission from 158, copyright 2020, Ivyspring International. (D) Schematic illustration of multicomponent Fmoc-FF/PLL-SH hydrogel nanoparticles, formed by co-assembling Fmoc-FF and PLL-SH to regulate immunosuppressive tumor microenvironment. Adapted with permission from 160, copyright 2017, American Chemical Society. (E) Schematic illustration of the co-assembled peptide-based treatment integrated by peptide DEAP-^D^PPA-1 and NLG919 for inhibiting Trp metabolism and blocking PD-L1against cancer cells. Adapted with permission from 162, copyright 2018, American Chemical Society.

In addition, peptides-drug co-assembled nanostructures can be developed for the delivery of immunotherapeutic agents. Cyclic diguanylate monophosphate (c-di-GMP) is a negatively charged stimulating factor of the interferon gene (STING) agonist with poor membrane permeability and bioavailability, which result in unfavorable therapeutic efficacy. To overcome this problem, Zeng et al. prepared a nanocomposite named c-di-GMP-PNT by co-assembling of Ac-KLVFFAL-NH_2_ (KL-7) and c-di-GMP, which demonstrated enhanced immune responses and preferred anti-tumor effect 156. Liu et al. described a GSH-responsive peptide derivative (Fbp-GDFDFDYD(E, S, or K)-ss-ERGD), which could co-assemble with antigens (endotoxin-free OVA) solution to form three different hydrogels (E-vac, S-vac, and K-vac) to deliver vaccine under the catalysis of GSH. *In vivo* results demonstrated that K-vac displayed the optimum production of antibodies and a more significant anti-tumor immune response among these three hydrogels*.* The co-assemblies could serve as vaccine delivery carriers, and the obtained immune effects could be tuned by changing the physical and chemical properties of peptides 157. In addition, their group also reported a Fbp-conjugated peptide derivative (Fbp-G^D^F^D^F^D^Y^D^K(γE)_2_-NH_2_, abbreviated as Comp. 1) that was able to deliver protein antigen efficiently. In this approach, ovalbumin (OVA) and hepatitis B surface antigen (HBsAg) were chosen as protein antigen models, which could induce Comp. 1 to assemble into nanofibers/hydrogels (Figure 9A-C). This vaccine platform showed multifunctional antitumor effects, including enhanced an-tumor immune activation effects by boosting antigen-presenting cell functions and promoting dendritic cell maturation, and diminished immune suppression *via* restraining COX-2/PEG2 pathway 158. Geng and co-workers designed a His_6_-metal assembly (HmA) delivery platform, which could co-assemble with various antibodies to form HmA@Abs for efficient intracellular delivery of antibodies 159.

Furthermore, peptide-based co-assemblies can kill tumor cells by regulating the immune system in two forms. Peptide assemblies exhibit anti-tumor properties by either activating anti-tumor immune cells or enhancing the effects of anti-tumor immunity. On the other hand, they have the potential to reduce the immunosuppressive effects and alter the microenvironments of tumors. An instance of this can be seen in the work by the Yan group, where they outlined a hydrogel co-assembled through electrostatic interactions batween poly-L-Lys (PLL) and Fmoc-FF. The finding demonstrated that this helical fibrous hydrogel could activate T cell response and effectively inhibit tumor growth (Figure 9D) 160.

#### 4.5.2. Immunotherapeutic agents-peptide co-assembly for combined immunotherapy

The combination of immunotherapy and other therapies, such as photodynamic therapy and chemotherapy, could greatly boost the immune response and improve the synergistic anti-tumor effects, which has drawn extensive attention. For example, Wang et al. constructed a co-assembled peptide vaccine by utilizing the co-assembly of two antigen epitope-conjugated peptides (ECPs), which showed enhanced anticancer T-cell responses. The combination of PD-L1/PD-1 inhibitor and IDO inhibitor showed an enhanced antitumor immunity 161. In this context, Nie et al described a multifunctional peptide derivative (DEAP-^D^PPA-1) consisting of isothiocyanate, MMP-metalloproteinase-2 responsive segment and PD-L1. The authors discovered that the micelle-like nanoparticles formed by the co-assembly of DEAP-^D^PPA-1 and NLG919 exhibited controlled drug release properties as a result of their dual-responsiveness in the tumor microenvironment (Figure 9E) 162. This superiority endowed the system with enhanced antitumor immune response and few side effects. Wang et al. utilized a functionalized amphiphilic peptide (Ac-CNYSKPTDRQYHFK(C18)GGPAK(C18) GADYKPITVKVN-NH_2_, PCP) for the co-delivery of Dox and R848 for combination immunotherapy. The combined assembling of PCP, Dox, and R848 facilitated the development of multi-agent prodrug (PCP@R848/DOX) that not only induces a immunogenic cell death (ICD), but also stimulates cytotoxic T lymphocytes, resulting in powerful synergistic anti-tumor immune responses, as per Yu et al.'s findings 163. Yu et al. designed three different peptides (AmpF, AmpFY, AmpFC919) based on pentapeptide FF-AmpF-FF(AmpF), all of which contained a central 4-aminoproline (AmpF) residue and two diphenylalanine (FF) fragments on both sides. AmpF was conjugated respectively with tyrosine and indoleamine 2, 3-dioxygenase (IDO) inhibitor (NLG919), AmpFY and AmpFC919 could be obtained. Once mixing AmpF, AmpFY, and AmpFC919, the co-assembled therapeutics AmpFY9 with pH shift nanostructures would be formed. *In vivo* assays revealed that AmpFY9 exhibited efficient delivery of NLG919 due to the augmented retention effects caused by pH-responsive morphological transformation. Interestingly, the additional γ-ray radiation would not only augment tumor immunogenicity but also stimulate immunity cytokine to inhibit the negative immune cells 164.

Overall, peptide-based co-assembly has made huge progress in remodeling tumor microenvironment, activating immune cells through ICD and developing cancer vaccines, which would expand the applicable scope of immunotherapy. With the emergence of immunotherapy combinations and artificial immune cells, the peptide-based co-assembly immunotherapy will eventually be more successful in readicating primary and metastatic tumor.

In a nutshell, peptide-based co-assembly has exhibited favorable prospects for application in chemotherapy, phototherapy, gene therapy, immunotherapy, radiotherapy, and combination therapy. As is known to all, some common functional groups found in peptide structures, such as carboxyl, hydroxyl, amino, etc, have the potential to interact with drugs* via* non-covalent interaction. The assembled peptides suitable for different drugs or certain anticancer drug should be adjusted according to different situations. For example, cisplatin is a common anticancer drug that could chelate with carboxyl groups in peptides. Thus, amino acids rich in carboxyl groups, such as glutamic acid, and aspartic acid were chosen as vital components of peptide segments. Some research groups represented by Yang et al. have synthesized NapFFYERGD, HCPT-FFERGD and Rh-GFFYERGD for co-assembling with cisplatin 103-105. In a word, when developing a peptide for a certain drug, it is necessary to incorporate the assembly sequence and appropriate functional sequence in the peptide in addition to introducing the specific sequence for interacting with the drugs. This process, which peptide co-assembles with drugs to form nanostructures with different morphologies, could be adjusted both *in vitro* and *in situ.* The drugs and peptides can co-assemble to speed up the assembly process, resulting in lower in MGC and CAC values, denser nanofibers, and stronger mechanical properties. In this co-assembled system, the drug release rate could be controlled flexibly by adjusting the ratio of peptides and drugs. Generally speaking, the drug can always exhibit sustained or controlled release profiles. The multifunctionality of peptides grants the multifaceted nature of the co-assembled system. For example, the conjugation of targeted groups could significantly enhance drug enrichment in tumors and result in stronger anti-cancer effects. The drug stability in the co-assembled system can be greatly improved by the incorporation of special functional groups. Besides, dug-peptide conjugates also could co-assemble with another drug to form nanostructures, achieving synergistic anticancer effects.

## 5. Conclusion and Perspective

This article summarized the research progress of peptide-based co-assembly in chemotherapy, phototherapy, gene therapy, immunotherapy, radiotherapy, and combination therapy. The co-assembly approach ensured a stable drug delivery system with elevated drug loading, tuned drug release profile, improved drug stability and enhanced targeting ability of the drug. In addition, peptide-based co-assembly has achieved remarkable progress in not only the improved therapeutic effects of tumor treatment but also the reduced toxic and side effects, which has been considered as one of the potential tumor therapeutic modalities.

Although significant advances have been achieved, these stated co-assembled delivery systems are still in the pre-clinical phases. The clinical application of peptide-based co-assembly is still challenged by several potential problems. To begin with, the stability of the co-assembled delivery system needs to be improved to reduce drug leakage and prolong systemic circulation time. The current strategies, such as PEGylation of peptides, utilization of D-type amino acids, or construction of composite peptide materials, have been considered desirable methods for improving the stability of peptides. Second, the pharmacokinetics of the co-assembled delivery system needs to be fully exploited by using unified quantitative standard methods. Besides, because of the heterogeneity of tumors, it should be an effective strategy for tumor therapy to use multiple therapeutic agents. What's more, to monitor the cellular uptake and the changes in tumor tissue, it is necessary to design novel integrated diagnosis and treatment models.

In addition, the lack of in-depth exploration of the precise mechanism of assembled nanostructures is still one of the main obstacles hindering the optimum design of the co-assembled delivery system. How to optimize the proportion of peptides and drugs to obtain co-assembled nanostructures with excellent stability and superior therapeutic effects is a major problem to be solved. With the development of computer simulation technology and supramolecular chemistry, more and more novel co-assembled delivery systems with controllable structures and sophisticated functions will be explored. We expect that the peptide-based co-assembly delivery system will serve as a bridge between nanotechnology and biotechnology, and open the door to innovative clinical uses for nanomedicine, playing a role in, not only anticancer, but also antibacterial, anti-inflammatory, and other aspects.

## Figures and Tables

**Table 1:** List of classification of co-assembled peptides and therapeutic agents for cancer therapy.

Classification of co-assembly	Peptide moieties	Active moieties	Factors triggering co-assembly	Cell lines	Ref
Chemotherapeutic agents-based co-assembly	ATKTA-S-S-ATKTA	Curcumin	Hydrogen bond	HeLa	98
	Nap-GFFYGRGDH	DOX	Electrostatic interaction	A549	85
	Ac-ATK(C18)DATGPAK(C18)TA	DOX	Hydrophobic interaction	PC-3	99
	Fmoc-FK (FK)/ Fmoc-FKK (FKK)	DOX	Aromatic interaction	MDA‐231	100
	CA-C11-GGGRGDS	MTX	Hydrogen bond	HeLa	88
	IDM-GFFYGRGDH	IMD+DOX	Electrostatic interaction	A549	101
	FKFEY-YSV	HCPT+ YSV	π-π stacking and electrostatic interactions	A549	102
	Nap-FFYERGD	Cisplatin+ Irinotecan	Coordination interaction	A549	103
	HCPT-FFERGD	HCPT+ Cisplatin	Coordination interaction	A549	104
	Rh-GFFYERGD	Rhe+ Cisplatin	Coordinated and hydrophobic interactions	A549	105
Radiosensitizer-based co-assembly	Npx-^D^F^D^F^D^E^D^Y	Cisplatin	Electrostatic interaction	A549	114
	Ce6-R_9_-^125^I-RGD	^125^I+Ce6+ miR-139-5p	Electrostatic interaction	HeLa	115
	NIA-D1	NIA+ R848	Non-covalent interaction	MC38	117
	FFRGD	H_2_S+IR	π-π stacking	H1299	118
Gene drugs-based co-assembly	Retro-Inverso CADY-K	siRNA	Hydrophobic interaction	U87	123
	KALA-2LMWP-NLS	pDNA	Electrostatic interaction	MCF-7	124
	TR4	pDNA	Non-covalent interactions	HeLa	125
	Fmoc-RRMEHRMEW	siRNA	Electrostatic interaction	HeLa	126
	Fmoc-RRMEHRMEW	AS1411	Non-covalent interaction	T47D	127
	GRVEVLYRGSW GRVRVLYRGSW	siRNA	Electrostatic interaction	MCF-7	128
	STR-H_16_R_8_	siRNA	Electrostatic interaction	HCT 116	129
	STR-HK	siRNA	Electrostatic interaction	A549	130
	TNCP	ASN	Electrostatic interaction	MDA-MB-231	131
	FC-PyTPA	Bcl-2 siRNA+PyTPA	Electrostatic interaction	HeLa	132
	Chol-HHHHHHH-AKRGARSTA	siRNA+1MT	Non-covalent interaction-	4T1	133
Phototherapeutic agents-based co-assembly	NapFFKYp	ICG	Coordinated intermolecular interaction	HeLa	136
	Z-Histidine-Obzl	Biliverdin	Hydrophobic, π-π and hydrogen bonding interaction	MCF7	137
	Fmoc-L_3_-OMe	m-THPP	π-π stacking and hydrophobic interaction	MCF-7	140
	H-FF-NH_2_·HCl, Fmoc-K	Chlorine6	Electrostatic, Hydrophobic, and π-π interaction	MCF7	92
	Fmoc-L-L	Chlorine6	Coordination, hydrophobic and π-π stacking interaction	MCF7	141
	Fmoc-L_3_-Arg	THPP	Hydrophobic, electrostatic, hydrogen bond and electrostatic interactions	4T1	144
	TAT-IR780	IR780+Dox	π-π stacking and hydrophobic interactions	4T1	145
	RKDVY(TP5)	TP5+ ICG	Non-covalent interaction	Pan02	146
	LND-K	TPPS_4_+LND	Electrostatic interaction	A375	147
Immunotherapeutic agents-based co-assembly	10 K-Adpgk	Adpgk	Electrostatic interaction	MC-38	154
	Epitope-R_8_	Epitope	Non-covalent interaction	B16-OVA	155
	AC-KLVFFAL-NH_2_	c-di-GMP	Electrostatic interaction	B16-F10	156
	Fbp-GDFDFDYD(E, S, or K)-ss-ERGD	OVA	Non-covalent interaction	E.G7-OVA	157
	Fbp-GDFDFDYDK(γE)_2_-NH_2_	OVA	Hydrogen bond and π-π interactions	E.G7-OVA	158
	HmA	Antibodies	Coordinative interaction	HeLa	159
	ECPs	K-OVA_257-264_+E-OVA_323-336_	Non-covalent interaction	E.G7-OVA	161
	DEAP-^D^PPA-1	^D^PPA +NLG919	Non-covalent interaction	B16-F10	162
	PCP	R848+DOX	Hydrophobic-hydrophilic interaction	4T1 luciferase	163
	AmpF, AmpFY, AmpFC919	NLG919+^125^I	Non-covalent interaction	4 T1	164

## Abbreviations

Nap: naphthylacetic acid
ZFF: carboxybenzyl-protected diphenylalanine
Fmoc: 9-Fluorenylmethoxycarbonyl
Boc: tert-butoxycarbonyl
PA: Peptide amphiphiles
R: arginine
A: alanine
D: aspartic acid
THP: pirarubicin
DOX: doxorubicin
MTX: methotrexate
MD: molecular dynamics
Lev: levofloxacin
CCM: curcumin
GSH: glutathione
IDM: indomethacin
YSV: tyroservatide
HCPT: hydroxycamptothecin
IRN: irinotecan
MMP-2: matrix metalloproteinase-2
Npx: naproxen
NIA: 2-(2-nitroimidazol-1-yl) acetic acid
DCs: dendritic cells
TLR: toll-like receptor
siRNA: small interfering RNA
pDNA: plasmid DNA
Apt: AS1411 aptamer
TPE: tetraphenylethene
1MT: 1-methyl-DL-tryptophan
PTT: photothermal therapy
PDT: photodynamic therapy
PTCAs: photothermal conversion agents
ALP: alkaline phosphatase
BV: biliverdin
Ce6: chlorine6
THPP: 5,10,15,20-Tetrakis (4-hydroxyphenyl) porphyrin
ICG: indocyanine green
LND: lonidamine
OVA: ovalbumin
HBsAg: hepatitis B surface antigen
PLL: poly-L-Lys
ECPs: epitope-conjugated peptides
ICD: immunogenic cell death
